# The role of alkyl substituents in deazaadenine-based diarylethene photoswitches

**DOI:** 10.3762/bjoc.12.106

**Published:** 2016-06-01

**Authors:** Christopher Sarter, Michael Heimes, Andres Jäschke

**Affiliations:** 1 Institut für Pharmazie und Molekulare Biotechnologie, Universität Heidelberg, 69120 Heidelberg, Germany

**Keywords:** diarylethene, nucleoside, electrocyclic rearrangement, photochromism, photoswitch

## Abstract

Diarylethenes are an important class of reversible photoswitches and often claimed to require two alkyl substituents at the carbon atoms between which the bond is formed or broken in the electrocyclic rearrangement. Here we probe this claim by the synthesis and characterization of four pairs of deazaadenine-based diarylethene photoswitches with either one or two methyl groups at these positions. Depending on the substitution pattern, diarylethenes with one alkyl group can exhibit significant photochromism, but they generally show poor stability towards extended UV irradiation, low thermal stability, and decreased fatigue resistance. The results obtained provide an important direction for the design of new efficient DNA photoswitches for the application in bionanotechnology and synthetic biology.

## Introduction

Most biomacromolecules are per se not responsive to light. Their conversion into photoresponsive molecules opens up new and innovative applications in materials sciences, bionanotechnology, synthetic biology, and biomedical diagnostics, and reversible photoswitching is a particularly attractive goal [1–12]. Light is a powerful and convenient trigger for manipulating structure and function, as it is non-invasive, provides high spatio-temporal resolution, and offers the option of orthogonality [13–14]. One of the common approaches for introducing photoresponsiveness into biomolecules is their covalent functionalization with small-molecule photoswitches [1–2,13,15–21]. Most reported applications use azobenzenes, while diarylethenes, spiropyrans, fulgides and hemithioindigos are applied less frequently [18–30].

**Figure 1:**
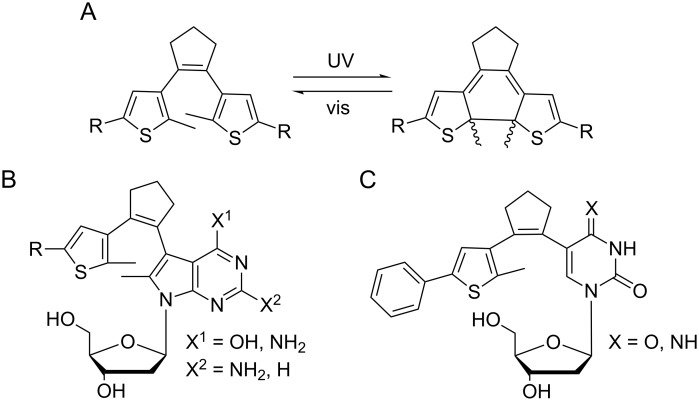
Diarylethene photoswitches. A: classical design and photoisomerization reaction [27]. B: purine-based photoswitches (1^st^ generation) [43–44], C: pyrimidine-based photoswitches (2^nd^ generation) [45].

Diarylethenes are an important class of photoswitches. Originally developed for optical information storage, they found many applications in materials sciences, nanotechnology, and biomimetic chemistry [31–42]. Their most typical architecture is shown in Figure 1A: two heteroaromatic, most often five-membered heterocyclic rings are connected via a cyclopentenyl bridge, which may also be perfluorinated. Substituents on the aryl rings tune the photophysical and chemical properties, such as absorption and isomerization wavelengths, thermal stability, and fatigue resistance.

In 2010 our group reported the first diarylethene-based photoswitchable nucleosides in which a purine nucleobase represented one of the aryl rings of the photoswitch (Figure 1B). Importantly and different from most other approaches towards photoswitchable nucleic acids, the nucleobase constituted an active part of the photoswitch (rather than an appendage), changing its bonding and hybridization upon switching [43–44]. This new class of diarylethenes showed promising photophysical and photochemical properties, such as the location of the photostationary states, reversibility of switching, thermal stability, and fatigue resistance.

In 2013 we developed a 2^nd^ generation of diarylethene-based photoswitchable nucleosides. The design of these molecules challenged two of the established design rules for diarylethene-based photoswitches (Figure 1C) [45]: (1) They incorporated a six-membered pyrimidine ring, and (2) – more importantly – only one methyl group, rather than two, was present at the positions where the bond is formed in the electrocyclic rearrangement. Still, these new diarylethenes had very favorable switching properties [45]. These results were surprising, considering that two alkyl groups at these positions are considered as essential for reversible switching throughout the published literature [27,39]. Experimental evidence for this requirement is, however, scarce. Few studies compare symmetrical switches with zero and two alkyl groups [39,46–47], but we were not able to locate a single publication where otherwise identical switches with one and two alkyl groups are compared.

Therefore, we set out to clarify this matter. If pyrimidine-based diarylethenes with one methyl group are good photoswitches, purine derivatives might work as well with just one methyl group. If this hypothesis was true, the synthesis of such photoswitches would be much simplified. Therefore, we report in the present communication the synthesis of four pairs of 7-deazaadenosine-based photoswitches with one or two methyl groups present, and their thorough characterization.

## Results and Discussion

### Design and synthesis of the photoswitches

**Scheme 1:**
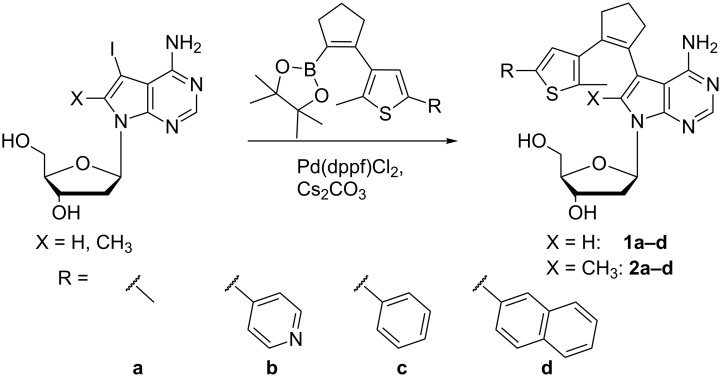
Synthesis of 7-deaza-2’-deoxyadenosine photoswitches with one and two methyl groups via Suzuki cross-coupling [45].

The design of the target compounds **1a–d** and **2a–d** is shown in Scheme 1. One aryl system is a 7-deaza-2’-deoxyadenosine, while the other one is a 2-methylthiophene derivatized with four different substituents in the 5-position. The only distinction between the two series of compounds is the absence (**1a–d**) or presence (**2a–d**) of a methyl group in position 8 of 7-deazaadenine. For the synthesis of these 8 compounds we used a convergent approach based on a central Suzuki cross-coupling reaction (Scheme 1).

**Scheme 2:**
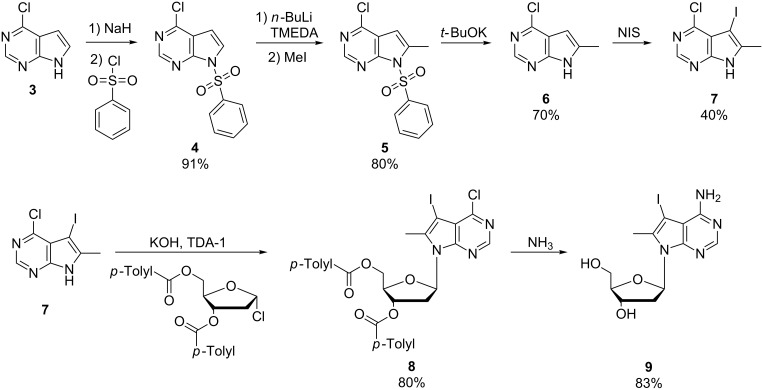
Optimized route for synthesis of 7-deaza-7-iodo-8-methyl-2’deoxyadenosine (**9**).

For the compounds with one methyl group (**1a–d**), 7-deaza-7-iodo-2’-deoxyadenosine was synthesized as described [48], while 7-deaza-7-iodo-8-methyl-2’-deoxyadenosine (**9**), required for the photoswitches with two methyl groups (**2a–d**), was synthesized using an optimized route based on our original work (Scheme 2) [43].

This route started from commercial 6-chloro-7-deazapurine and involved a transient protection of ring nitrogen N9 with benzenesulfonyl chloride, resulting in a four-fold increased yield of **6** compared to the original route.

**Scheme 3:**
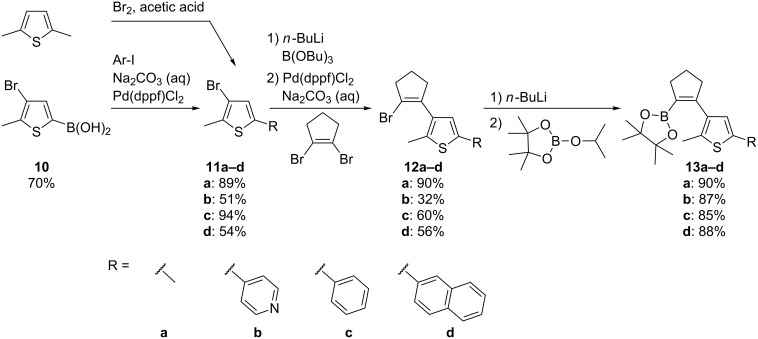
Synthesis of the boronic acid pinacolate esters.

The other part of the diarylethene, namely the cyclopentenyl bridge with the attached substituted thiophene ring was furnished as shown in Scheme 3 and activated as boronic acid pinacolate ester. Suzuki coupling yielded the eight target compounds in yields between 36% and 97%. Two of these compounds (**2b**,**c**) are identical to the ones reported previously [43], while the other 6 represent new compounds.

### Photophysical and photochemical characterization

**Figure 2:**
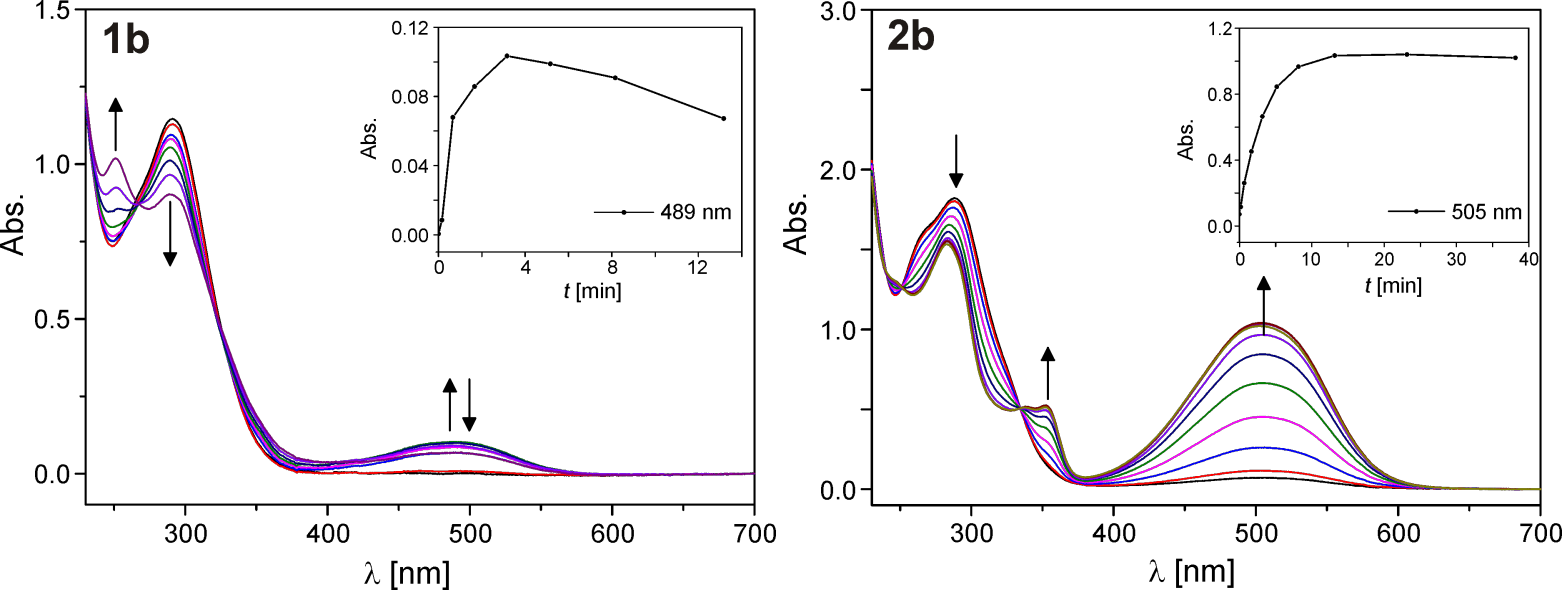
Spectral changes of the pyridyl switch with one (**1b**) and two (**2b**) methyl groups upon irradiation with UV light. A 60 µM solution of the compound in acetonitrile was irradiated for the indicated time intervals (see inset) with UV light (366 nm) and after each interval a UV–vis absorption spectrum was recorded.

For diarylethenes **1a–d** and **2a–d**, the photophysical and photochemical properties, in particular their photochromicity, switching kinetics and reversibility, fatigue resistance, and thermostability, were investigated. The main text shows only the analytical data for new pyridyl switch **1b** in comparison to the previously published and well-studied switch **2b** [43]; all other spectra are presented in Supporting Information File 1 Figures S1–S5. Figure 2 and Figure S1 (Supporting Information File 1) show the changes in the UV–vis spectra upon irradiation with UV light (366 nm, hand lamp) in acetonitrile.

Three differences are immediately apparent between the two classes of compounds: (1) At any given time point, the absorption cross-section is much higher with two methyl groups. To exclude solubility effects, we normalized the absorbance at the respective maximum of the closed-ring isomer to the 7-deazaadenosine absorbance at 280 nm of the open-ring form, yielding values between 0.08 and 0.35 for one (**1a–d**) and 0.25 to 0.67 for two (**2a–d**) methyl groups (Table 1). Among the compounds with one methyl group, only naphthyl derivative **1d** showed appreciable photochromicity (ratio 0.35). The normalized absorption cross-section is 1.75–6.73-fold higher for the two-methyl compounds. The strongest methyl group effect is observed for the pyridyl switches (Figure 2). (2) For all compounds, the absorption maximum of the closed-ring form is blue-shifted for the one-methyl compounds, in comparison to the two-methyl derivatives. This shift is between 14 (naphthyl, **1d** vs **2d**) and 50 nm (phenyl, **1c** vs **2c**, Table 1). (3) While all two-methyl compounds are stable towards further UV irradiation, the one-methyl compounds are not (see insets in the Figures). Absorbance decreases after reaching a maximum, implying that the closed-ring form undergoes a further light-dependent irreversible reaction. This decay is strongest for phenyl derivative **1c** which loses ~50% of its absorbance upon an irradiation of 1 hour. The same three tendencies can also be observed when the UV spectra are measured in ethanol and methanol (Supporting Information File 1, Figures S2 and S3), indicating that the differences between the one- and two-methyl switches are not due to solvent effects.

**Table 1:** Absorption characteristics of diarylethenes **1a–d** and **2a–d**.

R	λ_max,vis_ (**1a–d**)	A_λmax,vis_ /A_280 nm_ (**1a–d**)	λ_max,vis_ (**2a–d**)	A_λmax,vis_ /A_280 nm_ (**2a–d**)	Ratio^a^	Δλ_21_^b^

**a**	432 nm	0.08	447 nm	0.32	3.96	15
**b**	489 nm	0.10	505 nm	0.67	6.73	16
**c**	435 nm	0.14	485 nm	0.25	1.75	50
**d**	482 nm	0.35	496 nm	0.66	1.86	14

^a^(A_λmax,vis_/A_280nm_ (**2a–d**))/(A_λmax,vis_/A_280 nm_ (**1a–d**)). ^b^λ_max,vis_(**2a–d**) − λ_max,vis_(**1a–d**).

**Figure 3:**
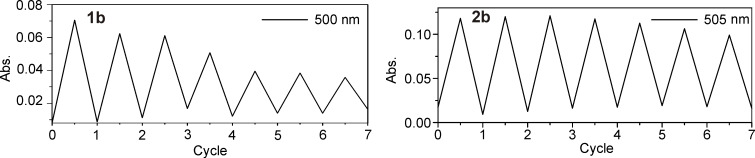
Reversibility measurements of deazaadenosine photoswitches with one and two methyl groups. A 60 µM solution of the compound in acetonitrile was irradiated with UV light until the (pseudo-)photostationary state was reached. After recording the UV–vis spectrum, the solution was irradiated with vis light for the same time. This procedure was repeated 7 times.

This latter observation already indicated poor reversibility and fatigue resistance for the one-methyl switches. We therefore undertook absorption measurements over multiple cycles of ring closure by UV and ring opening by vis irradiation only for pyridyl switch **1b**, which had shown the highest photostability (Figure 2). These measurements revealed almost complete loss of photoswitchability over 7 cycles, whereas the compound **2b** showed only slight deterioration (Figure 3). The more electron-rich two-methyl switches **2a,c** and **d** showed reduced reversibility, as also known for electron-rich classical diarylethenes (Supporting Information File 1, Figure S4) [49]. Interestingly, reversibility and fatigue resistance of the two-methyl switches are slightly improved in more polar solvents methanol and ethanol (Supporting Information File 1, Figure S5).

**Figure 4:**
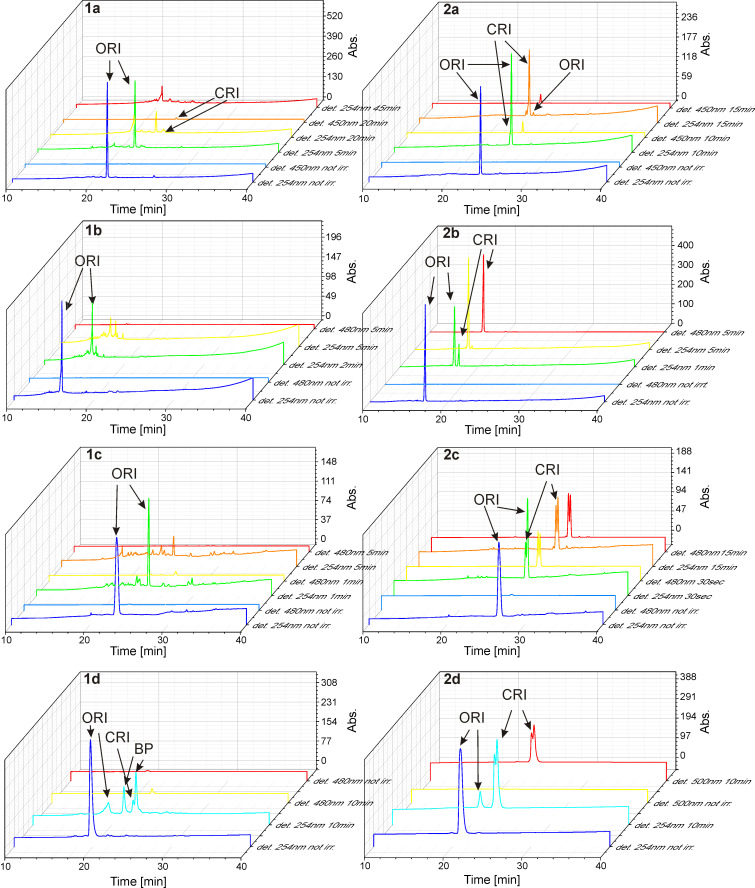
Stability analysis of compounds **1b–d** and **2b–d** upon different times of UV irradiation, monitored by HPLC with detection at 254 nm and at the absorption maximum of the closed-ring form. ORI: open-ring isomer, CRI: closed-ring isomer, BP: byproduct.

To understand why the one-methyl compounds are so rapidly degraded upon continued UV irradiation, we carried out HPLC and LC–MS analysis of solutions of **1b–d** after different times of UV irradiation and compared them with **2b–d** (Figure 4).

While no additional peaks appear in the chromatograms of the two-methyl compounds after reaching the photostationary state (where the closed-ring isomer dominates with more than 95% in all cases according to the integrated peak areas, Figure 4), the 1-methyl switches behave differently: For naphthyl switch **1d** the single peak corresponding to the open-ring form at 20 min (*m*/*z* of 539.21, strong absorption at 254 nm, no absorption at 520 nm) largely disappeared, whereas after extended UV irradiation three new peaks are detected by absorption at 254 nm, all with the same *m*/*z* value of 539.21 (Supporting Information File 1, Figure S6). One of them (at 22.2 min) also absorbs in the visible range, which is indicative of the closed-ring form. The other two peaks (at 21.2 and 22.8 min) are not colored and are much larger (in terms of integrated A_254_). These findings indicate that a large proportion of compound **1d** has undergone an irreversible side reaction and the colored closed-ring form is only a minor component after extended irradiation. The identical *m*/*z* values rule out additions or eliminations and instead suggest a rearrangement, such as the 1,2-dyotopic rearrangement described for diarylethenes [49–51]. This hypothesis is further supported by the appearance of two new signals in the NMR spectrum of an extensively irradiated solution of **1d** at δ = 2.53 and 2.71 (Supporting Information File 1, Figure S7), in agreement with values reported for isolated products of this rearrangement reaction [49]. Methyl switch **1a**, pyridyl switch **1b** and phenyl switch **1c**, on the other hand, decomposed into a multitude (>15) of different products, according to the HPLC chromatograms, indicating multiple degradation pathways. Due to this effect, the ratio between open- and closed-ring isomer in the pseudo-photostationary state cannot be determined.

**Figure 5:**
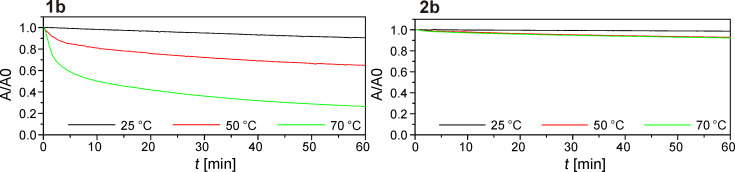
Thermostability measurements of the 7-deazaadenosine nucleosides. A 60 µM solution of the compound in acetonitrile was irradiated with UV light until the (pseudo)-photostationary state was reached. Then UV irradiation was switched off and the absorption at the visible light maximum of the closed ring form was measured over 1 h.

Finally, we analyzed the thermostability of the closed-ring form of the photoswitches. In all cases, the two-methyl compounds **2a–d** had higher thermostability. For the pyridyl switch (Figure 5), the two-methyl variant **2b** is absolutely stable at 25 °C and loses about 5% within 60 min at 50 or 70 °C, whereas the one-methyl version **1b** is already unstable at room temperature and rapidly degrades (40% in 4 min) at 70 °C. With the other three substituents, the same tendency can be observed (Supporting Information File 1, Figure S8).

## Conclusion

In the present study, we synthesized four pairs of substituted 7-deaza-2’-deoxyadenosine photoswitches bearing either one or two methyl groups at the position where the new bond is formed in the electrocyclic rearrangement. This investigation pursued two aims: i) we wanted to probe the common claim that two alkyl groups are required for photochromic diarylethenes by direct comparison, and ii) we wanted to test whether our prior successful violation of this “rule” with photochromic pyrimidine nucleosides could be expanded to purine nucleosides. Our analysis reveals that – depending on the substituents – the one-methyl 7-deaza-2’-deoxyadenosine compounds can indeed show photochromicity; in the case of a naphthyl substituent even to a significant extent. In all cases, however, the absorption cross-section was much smaller than for the 2-methyl compounds. More importantly, the one-methyl derivatives showed lower photostability, thermal stability, reversibility and fatigue resistance. Significant UV-irradiation-dependent side reactions were observed. From a practical point of view, one can therefore conclude that for these (i.e., deazaadenosine-based) photoswitches two methyl groups are more suitable than one. Overall, the results obtained provide an important direction for the design of new efficient photoswitches for application in bionanotechnology and synthetic biology.

## Supporting Information

File 1 Experimental part and additional spectra of investigated compounds.
