# Supplementary material for: The role of alkyl substituents in deazaadenine-based diarylethene photoswitches
Source: Beilstein J Org Chem. 2016 Jun 1;12:1103–10. doi: 10.3762/bjoc.12.106 (PMC4901873; doi:10.3762/bjoc.12.106)
Supplement: File 1 — Experimental part and additional spectra of investigated compounds. [file Beilstein_J_Org_Chem-12-1103-s001.pdf]

# Supporting Information

for

# The role of alkyl substituents in deazaadenine-based diarylethene photoswitches

Christopher Sarter, Michael Heimes and Andres Jäschke*§

Address: Institut für Pharmazie und Molekulare Biotechnologie, Universität Heidelberg, 69120 Heidelberg, Germany

Email: Andres Jäschke - jaeschke@uni-hd.de

§Homepage: http://www.jaeschke.uni-hd.de

*Corresponding author

Experimental part and additional spectra of investigated compounds

## Synthetic procedures

Commercially available reagents were purchased from Sigma Aldrich, Carbolution and Carbosynth. These reagents and laboratory grade solvents were used without further purification. Dry solvents were purchased in sealed bottles over molecular sieves. Solvents for oxygen-sensitive reactions were degassed prior use by a continuous Argon flow through the reaction mixture for about 10 minutes. All reactions were monitored by TLC (thin-layer chromatography) using coated silica plates with fluorescent indicator and visualized with UV-light (254 nm) or stained with a Blue-Shift solution ($Ce(SO_4)_2$; molybdatophosphoric acid; sulfuric acid). Reactions under high pressure were conducted in an autoclave (Büchi) equipped with a manometer or in microwave tubes sealed with a gas tight climber cap. Flash chromatography was performed according to Still et al. [1] with silica gel 60 (0.04–0.063 mm) using laboratory grade solvents. LC–MS experiments were performed on a Bruker microTOF-Q II ESI mass spectrometer connected to an Agilent 1200 Series HPLC system equipped with a multi-wavelength detector (MWD). A Phemonenex kinetex C18 column (2.6 µm, 100 × 2.1 mm) was used with a flow rate of 0.2 mL/min and eluting with a gradient of water and acetonitrile where 0.1% acetic acid was added. Analysis of the LC–MS measurements was performed using Hyphenation Star PP (Version 3.2.44.0) and Data Analysis (Version 4.0, SP 4) software (Bruker Daltonics). High-resolution mass spectra were recorded on a Bruker microTOF-Q II ESI (ESI). NMR-spectra were recorded on a Varian Systems 300 and 500 instrument. The chemical shifts (δ) are indicated in parts per million (ppm) downfield of TMS and referenced to the respective residual undeuterated solvent peak as follows: $CDCl_3$= 7.26 ppm; DMSO-$d_6$ = 2.50 ppm; MeOH-$d_4$ = 3.31 ppm for $^1$H NMR and $CDCl_3$ = 77.0 ppm; DMSO-$d_6$ = 39.4 ppm; MeOH-$d_4$ = 49.00 ppm for $^{13}$C NMR. Apparent coupling constants ($J$) are reported in Hz. HPLC measurements were performed using an Agilent 1100 Series HPLC system equipped with a multi-wavelength detector (MWD). A Phemonenex kinetex C18 column (Luna 2.5u C18(2)-HST, 100–3 mm) was used with a flow rate of 0.6 mL/min and eluting with a gradient of water and acetonitrile or buffer A and B (buffer A = 100 mM triethylammonium actetat in water, buffer B = 100 mM triethylammonium actetat in acetonitrile).

### 3,5-Dibromo-2-methylthiophene [2]

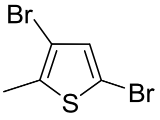

In a round-bottom flask, 2-methylthiophene (26.5 g, 0.224 mol) was dissolved in acetic acid (100 mL) and cooled down to 0 °C. A mixture of $Br_2$ (1.13mol, 32 mL) and acetic acid (40 mL) was added dropwise and the reaction mixture was stirred at 0 °C overnight. Distilled water (150 mL) and $Na_2CO_3$ were added until the pH of the mixture was neutral. The mixture was extracted three times with diethyl ether, the combined organic phases were washed with water and brine, dried over $MgSO_4$, filtered and the solvent was removed under reduced pressure. The black liquid residue was distilled under vacuum to afford a yellow liquid as product with a yield of 85%.

$^1$H-NMR (300 MHz, $CDCl_3$): δ = 6.85 (s, 1H), 2.34 (s, 3H, CH3). $^{13}$C-NMR (125 MHz, $CDCl_3$): δ = 136.0, 131.9, 108.6, 108.4, 14.8.

**4-Bromo-5-methylthiophene-2-ylboronic acid (10) [2]**

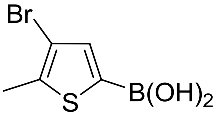


In a Schlenk flask under argon 3,5-dibromo-2-methylthiophene (12.8 g, 50 mmol) was dissolved in dry diethyl ether (240 mL) and degassed. The solution was cooled to −78 °C and *n*-BuLi (20.4 mL, 50 mmol, 2.5 M in hexane) was added dropwise. The mixture was stirred for 30 min at −78 °C.
Tributyl borate (12.68 g, 50 mmol) was added and the reaction mixture was stirred overnight. 1 M aq HCl (200 mL) was added, the diethyl ether phase was separated and extracted three times with 1 M aq NaOH solution (3 × 70 mL). To the combined NaOH phases conc. HCl was added until the mixture reached a pH of 1. In this acidic condition the product precipitates as a beige colored solid with a yield of 76%.

$^{1}$H-NMR (300 MHz, DMSO-d6): δ = 7.50 (s, 1H), 2.37 (s, 3H). $^{13}$C-NMR (125 MHz, DMSO-d6): δ = 139.7, 137.7, 109.5, 14.5. HRMS (ESI, positive) m/z: $[M+H]^+$ calculated for $[C_5H_6BBrO_2S]^+$: 222.9417, found: 222.9363.

**3-Bromo-2-methyl-5-phenylthiophene (11c) [2]**

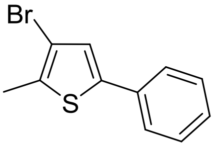


In a Schlenk flask under argon 4-bromo-5-methylthiophene-2-ylboronic acid (1.5 g, 6.8 mmol) was dissolved in THF (13 mL) and degassed. Iodbenzene (2.2 g, 8.2 mmol), Pd(dppf)$Cl_2$ (393 mg, 0.34 mmol) and aq $Na_2CO_3$ solution (20%, 13 mL) were added and the mixture was stirred under reflux overnight at 80 °C.
The reaction mixture was extracted three times with DCM, the combined organic phases were washed with water and brine, dried over $MgSO_4$, filtered and the solvents was removed under reduced pressure. Purification by flash column chromatography (pure cyclohexane) afforded a white solid with a yield of 94%.

$^{1}$H-NMR (300 MHz, $CDCl_3$): δ = 7.53-7.50 (m, 2H), 7.40-7.37 (m, 2H), 7.31-7.25 (m, 1H), 7.11(s, 1H), 2.42 (s, 3H). $^{13}$C-NMR (125 MHz, $CDCl_3$): δ = 141.3, 133.9, 135.1, 132.5, 129.1, 127.9, 125.7, 125.5, 121.9, 110.0, 15.0.

**4-(4-Bromo-5-methylthiophene-2-yl)pyridine (11b) [2]**

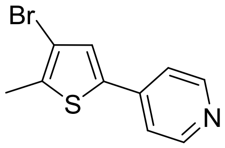

In a Schlenk flask under argon 4-bromo-5-methylthiophene-2-ylboronic acid (3.2 g, 14.14 mmol) was dissolved in THF (25 mL) and degassed. 4-bromopyridine hydrochlorid (4.43 g, 22.68 mmol), Pd(dppf)$Cl_2$ (740 mg, 0.91 mmol) and aq $Na_2CO_3$ solution (20%, 25 mL) were added and the mixture was stirred under reflux overnight at 80 °C. The reaction mixture was extracted three times with DCM, the combined organic phases were washed with water and brine, dried over $MgSO_4$, filtered and the solvents was removed under reduced pressure. Purification by flash column chromatography (pure cyclohexane + 1% $NEt_3$) afforded a brownish solid with a yield of 51%.

$^1$H-NMR (300 MHz, $CDCl_3$): δ = 8.59-8.57 (m, 2H), 7.39-7.37 (m, 2H), 7.31 (s, 1H), 2.44 (s, 3H). $^{13}$C-NMR (125 MHz, $CDCl_3$): δ = 150.3, 140.4, 137.7, 136.5, 127.9, 110.6, 109.9; 15.1. HRMS (ESI, positive) m/z: $[M+H]^+$ calculated for $[C_{10}H_8BrNS]^+$: 253.9634, found: 253.9626.

**3-Bromo-2-methyl-5-(naphthalen-2-yl)thiophene (11d)**

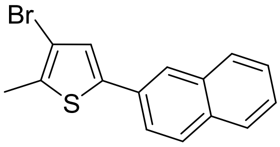

In a Schlenk flask under argon 4-bromo-5-methylthiophene-2-ylboronic acid (3 g, 13.58 mmol) was dissolved in THF (50 mL) and degassed. 2-bromonaphthalene (3.38 g, 16.3 mmol), Pd(dppf)$Cl_2$ (555 mg, 0.68 mmol) and aq. $Na_2CO_3$ solution (20%, 35 mL) were added and the mixture was stirred under reflux overnight at 80 °C. The reaction mixture was extracted three times with DCM, the combined organic phases were washed with water and brine, dried over $MgSO_4$, filtered and the solvents was removed under reduced pressure. Purification by flash column chromatography (pure cyclohexane) afforded a withe solid with a yield of 54%.

$^1$H-NMR (300 MHz, $CDCl_3$): δ = 7.95-7.94 (m, 1H), 7.85-7.80 (m, 3H), 7.66-7.62 (m, 1H), 7.52-7.43 (m, 2H), 7.240 (s, 1H), 2.45 (s, 3H). $^{13}$C-NMR (500 MHz, $CDCl_3$): δ = 141.1; 133.9; 133.5, 132.8, 130.8, 128.6, 128.0, 127.7, 126.6, 126.1, 125.9, 123.7, 123.6, 110.0, 14.9. HRMS (ESI, positive) m/z: $[M+H]^+$ calculated for $[C_{15}H_{11}BrS]^+$ : 302.9838, found: 302.9794.

**3-Bromo-2,5-dimethylthiophene (11a) [3]**

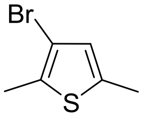

A mixture of $Br_2$ (8.55 g, 53.48 mmol) and acetic acid (22 mL) was added dropwise to a solution of 2,5-dimethylthiophene (5 g, 44.57 mmol) in acetic acid (33 mL). The reaction mixture was stirred in the dark overnight at rt. To the reaction mixture aq $Na_2CO_3$ solution (200 mL, 85g in 500 mL water) was added carefully. The water phase was extracted with diethyl ether three times. The combined organic phases were washed with water and brine, dried over $MgSO_4$, filtered and the solvent was removed under reduced pressure. Purification by flash column chromatography (pure cyclohexane) afforded a yellow liquid with a yield of 89%.

$^1$H-NMR (300 MHz, $CDCl_3$): δ = 6.55 (s, 1H), 2.40 (s, 3H), 2.32 (s, 3H). $^{13}$C-NMR (125 MHz, $CDCl_3$): δ = 135.8, 131.5, 127.5, 107.9, 15.3, 14.5.

**3-(2-Bromocyclopent-1-enyl)-2,5-dimethylthiophene (12a)**

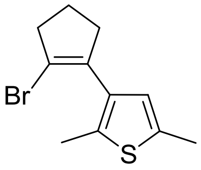


In a Schlenk flask under argon 3-bromo-2,5-dimethylthiophene (1 g, 5.23 mmol) was mixed with dry THF (30 mL) and degassed. The solution was cooled to −78 °C, *n*-BuLi (3 mL, 7.33 mmol, 2.5 M in hexane) was added dropwise and the mixture was stirred for 30 min at −78 °C.
Tributyl borate (1.93 g, 8.37 mmol) was added and the reaction mixture was stirred for 1 h at −78 °C. The solution was heated to rt, aq $Na_2CO_3$ solution (20%, 5 mL), Pd(dppf)$Cl_2$ (214 mg, 0.262 mmol) and 1,2 dibromocyclopentene (1.42 g, 6.28 mmol) were added. The mixture was heated to 75 °C and stirred under reflux overnight. The reaction mixture was extracted with ethyl acetate three times. The combined organic phases were washed with water and brine, dried over $MgSO_4$, filtered and the solvent was removed under reduced pressure. Purification by flash column chromatography (pure cyclohexane) afforded a yellowish oil with a yield of 90%.

$^1$H-NMR (300 MHz, $CDCl_3$): δ = 6.54 (s, 1H), 2.80-2.76 (m, 2H), 2.60-2.56 (m, 2H), 2.40 (s, 3H), 2.33 (s, 3H), 2.10-2.02 (m, 2H), $^{13}$C-NMR (125 MHz, $CDCl_3$): δ = 136.8, 135.1, 133.3, 125.5, 118.1, 110.0, 40.9, 36.8, 22.4, 15.2. HRMS (ESI, positive) m/z: $[M+H]^+$ calculated for $[C_{11}H_{13}BrS]^+$: 255.9921, found: 255.9920.

**3-(2-Bromocyclopent-1-enyl)-2-methyl-5-phenylthiophene (12c) [2]**

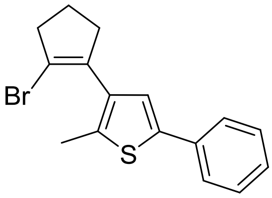


In a Schlenk flask under argon 3-bromo-2-methyl-5-phenylthiophene (3 g, 11.90 mmol) was dissolved in dry THF (75 mL) and degassed. The solution was cooled to −78 °C, *n*-BuLi (6.7 mL, 16.66 mmol, 2.5 M in hexane) was added dropwise and the mixture was stirred for 30 min at −78 °C.
Tributyl borate (4.35 g, 19.04 mmol) was added and the reaction mixture was stirred for 1 h at −78 °C. The solution was heated to rt, aq $Na_2CO_3$ solution (20%, 15mL), Pd(dppf)$Cl_2$ (486 mg, 0.595 mmol) and 1,2 dibromocyclopentene (4.36 g, 19.04 mmol) were added. The mixture was heated to 75°C and stirred under reflux overnight. The reaction mixture was extracted with ethyl acetate three times. The combined organic phases were washed with water and brine, dried over $MgSO_4$, filtered and the solvent was removed under reduced pressure. Purification by flash column chromatography (pure cyclohexane) afforded a white solid with a yield of 60%.

$^1$H-NMR (300 MHz, $CDCl_3$): δ = 7.72-7.67 (m, 2H), 7.52-7.47 (m, 2H), 7.41-7.36 (m, 1H), 7.28 (s, 1H), 3.00-2.93 (m, 2H), 2.83-2.77 (m, 2H), 2.57 (s, 3H), 2.28-2.17 (m, 2H). $^{13}$C-NMR (125 MHz, $CDCl_3$): δ = 140.0, 136.5, 135.4, 134.4, 134.3, 128.3, 127.1, 125.4, 123.6, 118.8, 40.9, 36.8, 22.4, 14.9.

**4-(4-(2-Bromocyclopent-1-enyl)-5-methylthiophen-2-yl)pyridine (12b) [2]**

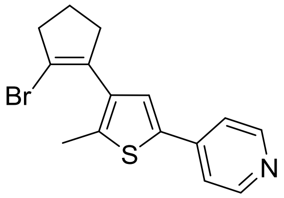

In a Schlenk flask under argon 4-(4-bromo-5-methylthiophen-2-yl)pyridine (1.2 g, 4.72 mmol) was dissolved in dry THF (25 mL) and degassed. The solution was cooled to -78°C, *n*-BuLi (2.7 mL, 6.6 mmol, 2.5 M in hexane) was added dropwise and the mixture was stirred for 30 min at −78 °C. Tributyl borate (1.7 g, 7.55 mmol) was added and the reaction mixture was stirred for 1 h at −78 °C. The solution was heated to rt, aq $Na_2CO_3$ solution (20%, 5 mL), Pd(dppf)$Cl_2$ (193 mg, 0.236 mmol) and 1,2 dibromocyclopentene (1.33 g, 5.66 mmol) were added. The mixture was heated to 75 °C and stirred under reflux overnight. The reaction mixture was extracted with ethyl acetate three times. The combined organic phases were washed with water and brine, dried over $MgSO_4$, filtered and the solvent was removed under reduced pressure. Purification by flash column chromatography (pure cyclohexane + 1% $NEt_3$) afforded a brownish oil with a yield of 32%.

1H-NMR (300 MHz, CDCl3): δ = 8.56-8.54 (m, 2H), 7.41-7.39 (m, 2H), 7.33 (s, 1H), 2.85-2.79 (m, 2H), 2.68-2.61 (m, 2H), 2.44 (s, 3H), 2.14-2.06 (m, 2H). $^{13}$C-NMR (500 MHz, $CDCl_3$): δ = 150.1, 141.4, 138.3, 136.7, 135.9, 135.1, 126.1, 119.6, 119.3, 40.9, 36.8, 22.4, 15.1. HRMS (ESI, positive) m/z: $[M+H]^+$ calculated for $[C_{15}H_{14}BrNS]^+$: 322.0083, found: 322.0087.

**3-(2-Bromocyclopent-1-enyl)-2-methyl-5-(naphthalen-2-yl)thiophene (12d)**

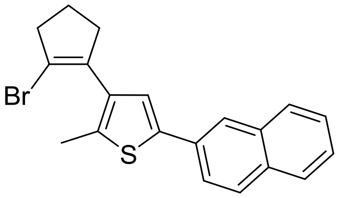

In a Schlenk flask under argon 3-bromo-2-methyl-5-(naphthalen-2-yl)thiophene (500 mg, 1.65 mmol) was dissolved in dry THF (15 mL) and degassed. The solution was cooled to −78 °C, *n*-BuLi (1 mL, 2.31 mmol, 2.5 M in hexane) was added dropwise and the mixture was stirred for 30 min at −78 °C. Tributyl borate (608 mg, 2.64 mmol) was added and the reaction mixture was stirred for 1 h at -78°C. The solution was heated to rt, aq $Na_2CO_3$ solution (20%, 3 mL), Pd(dppf)$Cl_2$ (68 mg, 825 µmol) and 1,2 dibromocyclopentene (447 mg, 1.98 mmol) were added. The mixture was heated to 75 °C and stirred under reflux overnight. Reaction mixture was extracted with ethyl acetate three times. The combined organic phases were washed with water and brine, dried over $MgSO_4$, filtered and the solvent was removed under reduced pressure. Purification by flash column chromatography (pure cyclohexane) afforded a white solid with a yield of 56%.

$^1$H-NMR (300 MHz, $CDCl_3$): δ = 7.97 (s, 1H), 7.83-7.79 (m, 3H), 7.72-7.67 (m, 1H), 7.50-7.40 (m, 2H), 7.25 (s, 1H), 2.87-2.79 (m, 2H), 2.72-2.65 (m, 2H), 2.46 (s, 3H), 2.15-2.05 (m, 2H), $^{13}$C-NMR (500 MHz, $CDCl_3$): δ = 140.1, 136.5, 135.7, 134.6, 133.6, 132.5, 131.7, 128.4, 127.9, 127.7, 126.4, 125.7, 124.0, 123.6, 123.3, 118.9, 40.9, 36.9, 22.5, 15.0. HRMS (ESI, positive) m/z: $[M+Na]^+$ calculated for $[C_{20}H_{17}BrNaS]^+$: 391.0126, found: 391.0127.

**2-(2-(2,5-Dimethylthiophen-3-yl)cyclopent-1-enyl)-4,4,5,5-tetramethyl-1,3,2-dioxaborolane (13a)**

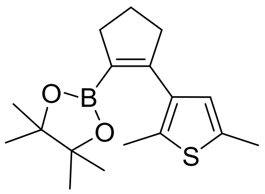

In a Schlenk flask under argon atmosphere 3-(2-bromocyclopent-1-enyl)-2,5-dimethylthiophene (640 mg, 2.5 mmol) was dissolved in dry THF (20 mL) and degassed. The solution was cooled to −78 °C, *n*-BuLi (1.4 mL, 3.5 mmol, 2.5 M in hexane) was added dropwise and the mixture was stirred for 30 min at −78 °C.
2-Isopropoxy-4,4,5,5-tetramethyl-1,3,2-dioxaborolane (651 mg, 3.5 mmol) was added and the reaction mixture was stirred for 1 h at −78 °C. Reaction mixture was heated to rt and extracted with ethyl acetate three times. The combined organic phases were washed with water and brine, dried over $MgSO_4$, filtered and the solvent was removed under reduced pressure. Purification by flash column chromatography (cyclohexane/ethyl acetate 20:1) afforded a yellow oil with a yield of 90%.

$^{1}$H-NMR (300 MHz, $CDCl_3$): δ = 6.52 (s, 1H), 2.66-2.57 (m, 4H), 2.37 (s, 3H), 2.30 (s, 3H), 1.94-1.88 (m, 2H). $^{13}$C-NMR (125 MHz, $CDCl_3$): δ = 152.1, 138.4, 136.1, 133.9, 127.1, 82.7, 40.1, 37.0, 24.7, 22.9, 15.0. HRMS (ESI, positive) m/z: $[M+Na]^+$ calculated for $[C_{17}H_{25}NaBO_2S]^+$: 327.1564, found: 327.1566.

**4,4,5,5-Tetramethyl-2-(2-(2-methyl-5-phenylthiophen-3-yl)cyclopent-1-enyl)-1,3,2-dioxaborolane (13c) [2]**

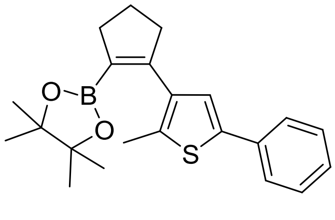

In a Schlenk flask under argon atmosphere 3-(2-bromocyclopent-1-enyl)-2-methyl-5-phenylthiophene (1 g, 3.13 mmol) was dissolved in dry THF (20 mL) and degassed. The solution was cooled to −78 °C, *n*-BuLi (1.76 mL, 4.4 mmol, 2.5 M in hexane) was added dropwise and the mixture was stirred for 30 min at −78 °C.
2-Isopropoxy-4,4,5,5-tetramethyl-1,3,2-dioxaborolane (820 mg, 4.4 mmol) was added and the reaction mixture was stirred for 1 h at −78 °C. Reaction mixture was heated to rt and extracted with ethyl acetate three times. The combined organic phases were washed with water and brine, dried over $MgSO_4$, filtered and the solvent was removed under reduced pressure. Purification by flash column chromatography (cyclohexane/ethyl acetate 20:1) afforded a white solid with a yield of 85%.

$^{1}$H-NMR (300 MHz, $CDCl_3$): δ = 7.57-7.53 (m, 2H), 7.37-7.32 (m, 2H), 7.24-7.17 (m, 1H), 7.14 (s, 1H), 2.71-2.62 (m, 4H), 2.39 (s, 3H), 2.01-1.88 (m, 2H), 1.19 (s, 12H). $^{13}$C-NMR (125 MHz, $CDCl_3$): δ = 152.0, 138.4, 137.6, 134.7, 134.4, 128.7, 128.7, 126.7, 125.1, 82.8, 40.2, 37.1, 24.8, 24.3, 14.6. HRMS (ESI, positive) m/z: $[M+Na]^+$ calculated for $[C_{22}H_{27}BNaNO_2S]^+$: 389.1721, found: 389.1718.

**4-(5-Methyl-4-(2-(4,4,5,5-tetramethyl-1,3,2-dioxaborolan-2-yl)cyclopent-1-enyl)thiophen-2-yl)pyridine (13b) [2]**

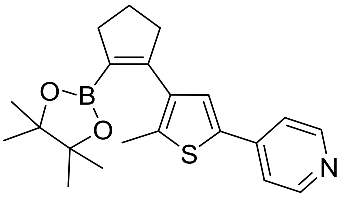


In a Schlenk flask under argon atmosphere 4-(4-(2-bromocyclopent-1-enyl)-5-methylthiophen-2-yl)pyridine (300 mg, 0.94 mmol) was dissolved in dry THF (10 mL) and degassed. The solution was cooled to −78 °C , *n*-BuLi (0.5 mL, 1.31 mmol, 2.5 M in hexane) was added dropwise and the mixture was stirred for 30 min at −78 °C.
2-Isopropoxy-4,4,5,5-tetramethyl-1,3,2-dioxaborolane (244 mg, 1.31 mmol) was added and the reaction mixture was stirred for 1 h at −78 °C. Reaction mixture was heated to rt and extracted with ethyl acetate three times. The combined organic phases were washed with water and brine, dried over $MgSO_4$, filtered and the solvent was removed under reduced pressure. Purification by flash column chromatography (cyclohexane/ethyl acetate 20:1+ 1%$NEt_3$) afforded a brown oil with a yield of 87%.

$^1$H-NMR (300 MHz, $CDCl_3$): δ = 8.55-8.52 (m, 2H), 7.43-7.41 (m, 2H), 7.36 (s, 1H), 2.70-2.62 (m, 4H), 2.41 (s, 3H), 1.99-1.92 (m, 2H), 1.18 (s, 12H). HRMS (ESI, positive) m/z: $[M+H]^+$ calculated for $[C_{21}H_{26}BNO_2S]^+$: 368.1854, found: 368.1841.

**4,4,5,5-Tetramethyl-2-(2-(2-methyl-5-(naphthalen-2-yl)thiophen-3-yl)cyclopent-1-enyl)-1,3,2-dioxaborolane (13d)**

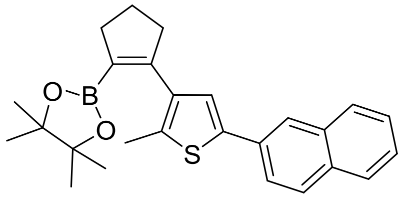


In a Schlenk flask under argon atmosphere 3-(2-bromocyclopent-1-enyl)-2-methyl-5-(naphthalen-2-yl)thiophene (300 mg, 0.81 mmol) was dissolved in dry THF (10 mL) and degassed. The solution was cooled to −78 °C, *n*-BuLi (0.53 mL, 1.14 mmol, 2.5 M in hexane) was added drop wise and the mixture was stirred for 30 min at −78 °C.
2-Isopropoxy-4,4,5,5-tetramethyl-1,3,2-dioxaborolane (212 mg, 1.14 mmol) was added and the reaction mixture was stirred for 1 h at −78 °C. Reaction mixture was heated to rt and extracted with ethyl acetate three times. The combined organic phases were washed with water and brine, dried over $MgSO_4$, filtered and the solvent was removed under reduced pressure. Purification by flash column chromatography (cyclohexane/ethyl acetate 20:1) afforded a white solid with a yield of 88%.

$^1$H-NMR (300 MHz, $CDCl_3$): δ = 7.97 (s, 1H), 7.84-7.78 (m, 3H), 7.72-7.68 (m, 1H), 7.50-7.41 (m, 2H), 7.28 (s, 1H), 2.75-2.63 (m, 4H), 2.43 (s, 3H), 2.00-1.93 (m, 2H), 1.20 (s, 12). $^{13}$C-NMR (125 MHz, $CDCl_3$): δ = 152.0, 137.7, 135.7, 134.7, 133.7, 132.5, 131.9, 128.4, 127.9, 127.6, 126.4, 125.7. 124.1, 123.5, 123.3, 83.0, 40.2, 37.1, 24.8, 23.4, 14.7. HRMS (ESI, positive) m/z: $[M+Na]^+$ calculated for $[C_{26}H_{29}BNaO_2S]^+$: 439.1878, found: 439.1886.

**4-Chloro-7-(phenylsulfonyl)-7*H*-pyrrolo[2,3-*d*]pyrimidine (4) [4]**

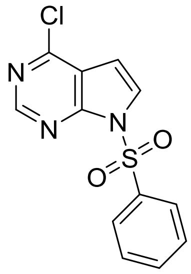


In a Schlenk flask under argon 6-chloro-7-deazapurine (5 g, 32 mmol) was dissolved in dry THF (150 mL) and cooled to 0 °C. NaH (1.94 g, 80 mmol) was added carefully and stirred for 3 h. Benzenesulfonyl chloride (8.6 g, 48 mmol) was added and the mixture was stirred overnight at rt. The mixture was mixed with aq $NH_4Cl$ (300 mL), extracted with ethyl acetate three times, washed with water and brine, dried over $MgSO_4$ and filtered. Cyclohexane was added to the solution to crystalize the product as a white solid with a yield of 91%.

$^1$H-NMR (300 MHz, DMSO-d$_6$): δ = 8.82 (s, 1H), 8.19-8.17 (m, 1H), 8.16-8.13 (m, 2H), 7.82-7.76 (m, 1H), 7.71-7.64 (m, 2H), 6.98 (d, *J* = 4,1Hz, 1H). $^{13}$C-NMR (125 MHz, DMSO-d$_6$): δ = 152.6, 152.1, 150.6, 136.6, 135.5, 129.9, 128.6, 127.8, 119.2, 103.4. HRMS (ESI, positive) m/z: $[M+H]^+$ calculated for $[C_{12}H_8ClN_3O_2S]^+$: 294.0102, found: 294.0104.

**4-Chloro-6-methyl-7-(phenylsulfonyl)-7*H*-pyrrolo[2,3-*d*]pyrimidine (5) [4]**

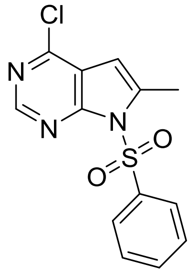


In a Schlenk flask under argon atmosphere 4-chloro-7-(phenylsulfonyl)-7*H*-pyrrolo[2,3-*d*]pyrimidine (8 g, 27 mmol) was dissolved in dry THF (200 mL) and cooled to −78 °C. TMEDA (5.2 g, 45 mmol) and *n*-BuLi (18.4 mL, 43 mmol, 2.5 M in hexane) were added dropwise at the same time, then MeI (8.8 mL, 137 mmol) was added and the reaction mixture was heated to rt and stirred overnight. The mixture was mixed with aq $NH_4Cl$ (300 mL), extracted with ethyl acetate three times, washed with water and brine, dried over $MgSO_4$ and filtered. Purification by flash column chromatography (cyclohexane/ethyl acetate 4:1) afforded **5** as a white solid in a yield of 80%.

$^1$H-NMR (300 MHz, DMSO-d$_6$): δ = 8.74 (s, 1H), 8.18-8.13 (m, 2H), 7.80-7.70 (m, 1H), 7.68-7.62 (m, 2H), 6.75 (s, 1H). $^1$H-NMR (125 MHz, DMSO-d$_6$): 152.6, 152.1, 150.6, 136.6, 135.5, 129.9, 128.6, 127.8, 119.2, 103.4. HRMS (ESI, positive) m/z: $[M+H]^+$ calculated for $[C_{13}H_{10}ClN_3O_2S]^+$: 308.0201, found: 308.0200.

**4-Chloro-6-methyl-7*H*-pyrrolo[2,3-*d*]pyrimidine (6)[2]**

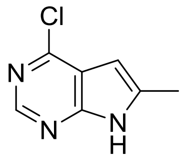


4-Chloro-6-methyl-7-(phenylsulfonyl)-7*H*-pyrrolo[2,3-*d*]pyrimidine (3,35 g,10,9 mmol) was dissolved in THF, potassium *tert*-butoxide (6.12 g, 54,6 mmol) was added and the mixture was stirred for 3 h at rt. $NaHCO_3$ (100 mL) was added and the mixture was extracted with ethyl acetate three times, washed with water and brine, dried over $MgSO_4$ and filtered. Purification by flash column chromatography (cyclohexane/ethyl acetate 4:1) afforded **6** as a white solid in a yield of 70%.

$^1$H-NMR (300 MHz, DMSO-d$_6$): δ = 12.39 (s, 1H), 8.48 (s, 1H), 6.31-6.30 (m, 1H), 3.51 (s, 3H).

**4-Chloro-5-iodo-6-methyl-7*H*-pyrrolo[2,3-*d*]pyrimidine (7) [2]**

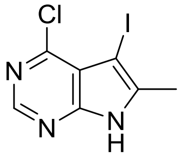


To a suspension of 4-chloro-6-methyl-7*H*-pyrrolo[2,3-d]pyrimidine (1.75 g, 10.5 mmol) in DCM (116 mL) *N*-iodosuccinimide (2.83 g, 12.6 mmol) was added and stirred for 2 h at rt. Filtration and washing with DCM afforded **7** as a white solid with a yield of 40%.

$^1$H-NMR (300 MHz, DMSO-d$_6$): δ = 12.92 (s, 1H), 8.51 (s, 1H), 2.42 (s, 3H). $^{13}$C-NMR (125 MHz, DMSO-d$_6$): δ = 151.90, 149.49, 148.86, 141.53, 116.63, 53.12, 14.62. HRMS (ESI) m/z: [M + Na]$^+$ calculated for $[C_7H_5ClN_3NaI]^+$: 315.9109, found: 315.9111.

**(2*R*,3*S*,5*R*)-5-(4-Chloro-5-iodo-6-methyl-7*H*-pyrrolo[2,3-*d*]pyrimidin-7-yl)-2-(((4-methylbenzoyl)oxy)methyl)tetrahydrofuran-3-yl 4-methylbenzoate (8) [2]**

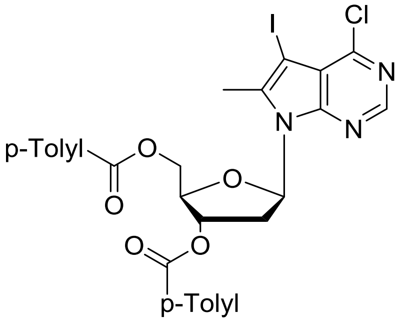


In a Schlenk flask under argon 4-chloro-5-iodo-6-methyl-7*H*-pyrrolo-[2,3-*d*]pyrimidine (1.09 g, 3.72 mmol) and KOH (837 mg, 13.0 mmol) were dissolved in acetonitrile (45 mL). Tris[2-(2-methoxyethoxy)ethyl]-amin (TDA-1) (149 µL, 0.620 mmol) and (2*R*,3*S*,5*S*)-5-chloro-2-(((4-methylbenzoyl)oxy)methyl)tetrahydro-furan-3-yl 4-methylbenzoat (1.88 g, 4.82 mmol) was added and the mixture was stirred for 90 min at rt. Purification by flash column chromatography (cyclohexane/ethyl acetate 4:1) afforded **8** as a white solid with a yield of 80%.

$^1$H-NMR (300 MHz, $CDCl_3$): δ = 8.48 (s, 1H); 7.97 (d, J = 8.2 Hz, 2H); 7.86 (d, J = 8.2 Hz, 2H); 7.28 (d, J = 8.0 Hz, 2H), 7.20 (d, J = 8.0 Hz, 2H), 6.56 (t, J = 7.2 Hz, 1H), 5.95 (dt, J = 7.3, 3.7 Hz, 1H), 4.80 (dd, J = 11.8, 3.9 Hz, 1H), 4.64 – 4.50 (m, 3H), 3.75 (dt, J = 14.6, 7.5 Hz, 1H), 2.63 (s, 3H), 2.44 (s, 3H), 2.41 (s, 3H). $^{13}$C-NMR (125 MHz, $CDCl_3$): δ = 166.33, 151.43, 144.55, 144.10, 141.45, 129.95, 129.81, 129.42, 129.26, 126.95, 117.72, 85.04, 82.09, 74.67, 63.65, 56.97, 36.11, 21.90, 21.85, 15.25. HRMS (ESI) m/z: [M + Na]$^+$ calculated for $[C_{28}H_{25}ClIN_3O_5Na]^+$: 668.0420, found: 668.0407.

**(2*R*,3*S*,5*R*)-5-(4-Amino-5-iodo-6-methyl-7*H*-pyrrolo[2,3-*d*]pyrimidin-7-yl)-2-(hydroxymethyl)tetrahydrofuran-3-ol (9)**

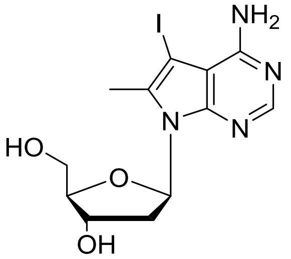


In an autoclave (2*R*,3*S*,5*R*)-5-(4-chloro-5-iodo-6-methyl-7*H*-pyrrolo-[2,3-*d*]pyrimidin-7-yl)-2-(((4-methylbenzoyl)oxy)methyl)tetrahydrofuran-3-yl 4-methylbenzoate (516 mg, 0.800 mmol) was dissolved in a mixture of aq ammonia solution (3.20 mL, 25%) and dioxane (3.20 mL). The reaction mixture was stirred at 5 bar and 110 °C for 2 days. The mixture was coevaporated with toluene 3 times. Purification by flash column chromatography (DCM/MeOH 20:1) afforded **9** as a white solid with a yield of 83%.

$^1$H-NMR (300 MHz, DMSO-d$_6$): δ = 8.04 (s, 1H), 6.65 (s, 2H), 6.43 (dd, J = 8.5; 6.5 Hz, 1H), 4.39 (s, 1H), 3.81 (dd, J = 7.2, 4.0 Hz, 1H), 3.68 – 3.57 (m, 1H), 3.57 – 3.45 (m, 1H), 3.31 (s, 2H), 2.94 – 2.83 (m, 1H), 2.43 (s, 3H), 2.12 – 2.03 (m, 1H). $^{13}$C-NMR (125 MHz, DMSO-d$_6$): δ = 156.37, 150.53, 150.03, 134.53, 102.99, 87.28, 84.20, 70.79, 61.85, 55.37, 38.26, 13.88. HRMS (ESI) m/z: [M + H]$^+$ calculated for [$C_{12}H_{16}IN_4O_3$]$^+$: 391.0262, found: 391.0266.

**(2*R*,3*S*,5*R*)-5-(4-Amino-5-(2-(2,5-dimethylthiophen-3-yl)cyclopent-1-en-1-yl)-7*H*-pyrrolo[2,3-*d*]pyrimidin-7-yl)-2-(hydroxymethyl)tetrahydrofuran-3-ol (1a)**

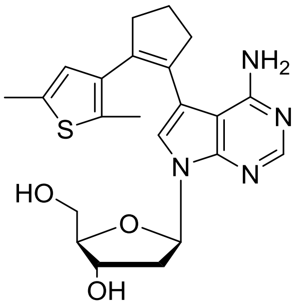


In a microwave vial (2*R*,3*S*,5*R*)-5-(4-amino-5-iodo-6-methyl-7*H*-pyrrolo[2,3-*d*]pyrimidin-7-yl)-2-(hydroxymethyl)tetrahydrofuran-3-ol (50 mg, 0.134 mmol), 2-(2,5-dimethylthiophen-3-yl)-4,4,5,5-tetramethyl-1,3,2-dioxaborolane (82.0 mg,0.268 mmol), Pd(dppf)$Cl_2$ (6 mg, 6.7 µmol) and $Cs_2CO_3$ (217 mg, 0.67 mmol) were dissolved in acetonitrile (2 mL) and water (1 mL) under argon. The mixture was degassed 3 times and stirred at 120 °C for 1 h. The reaction mixture was absorbed on silica and purified by flash column chromatography (silica gel, DCM/MeOH 10:1) afforded **1a** as yellowish solid with a yield of 46%.

$^1$H-NMR (300 MHz, $CD_3OD$): δ = 7.99 (s, 1H), 7.63 – 7.47 (m, 1H), 7.25 (s, 1H), 6.52 – 6.46 (m, 1H), 4.54 – 4.47 (m, 1H), 4.01 (dd, J = 6.0, 3.3 Hz, 1H), 3.84 – 3.65 (m, 2H), 2.86 (dd, J = 12.7, 6.1 Hz, 4H), 2.63 (ddd, J = 13.8, 8.1, 6.0 Hz, 1H), 2.34 – 2.29 (m, 1H), 2.26 (s, 3H), 2.15 – 2.03 (m, 2H), 1.94 (s, 3H). $^{13}$C-NMR (125 MHz, $CD_3OD$): δ = 158.96, 151.96, 150.35, 137.04, 136.58, 135.37, 133.98, 133.21, 127.43, 122.55, 115.07, 103.44, 89.02, 86.58, 73.10, 63.79, 41.40, 41.14, 39.66, 23.70, 14.98, 14.57. HRMS (ESI) m/z: [M + H]$^+$ calculated for [$C_{22}H_{27}N_4O_3S$]$^+$: 427.1826, found: 427.1792.

**(2*R*,3*S*,5*R*)-5-(4-Amino-5-(2-(2-methyl-5-phenylthiophen-3-yl)cyclopent-1-en-1-yl)-7*H*-pyrrolo[2,3-*d*]pyrimidin-7-yl)-2-(hydroxymethyl)tetrahydrofuran-3-ol (1c)**

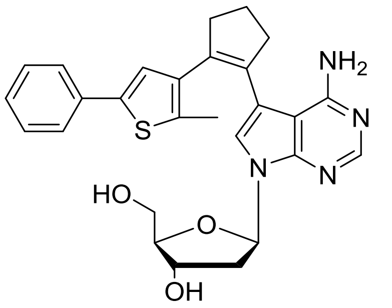


In a microwave vial 2*R*,3*S*,5*R*)-5-(4-amino-5-iodo-6-methyl-7*H*-pyrrolo[2,3-*d*]pyrimidin-7-yl)-2-(hydroxymethyl)tetrahydrofuran-3-ol (50 mg, 0.134 mmol), 4,4,5,5-tetramethyl-2-(2-(2-methyl-5-phenylthiophen-3-yl)cyclopent-1-en-1-yl)-1,3,2-dioxaborolan (94.0 mg, 0.268 mmol), Pd(dppf)$Cl_2$ (6 mg, 6.7 µmol) and $Cs_2CO_3$ (217 mg, 0.67 mmol) were dissolved in acetonitrile (2 mL) and water (1 mL) under argon. The mixture was degassed 3 times and stirred at 120 °C for 1 h. The reaction mixture was absorbed on silica and purified by flash column chromatography (silica gel, DCM/MeOH 10:1) affording **1b** as yellowish solid in a yield of 75%.

$^1$H-NMR (300 MHz, $CD_3OD$): δ = 7.96 (s, 1H), 7.36 – 7.22 (m, 6H), 7.03 (s, 1H), 6.51 – 6.45 (m, 1H), 4.52 – 4.46 (m, 1H), 4.00 (d, J = 2.7 Hz, 1H), 3.86 – 3.70 (m, 2H), 2.95 – 2.83 (m, 4H), 2.49 – 2.41 (m, 1H), 2.32 – 2.23 (m, 1H), 2.14 – 2.07 (m, 2H), 2.02 (s, 3H). $^{13}$C-NMR (125 MHz, $CD_3OD$): δ = 158.93, 152.08, 150.42, 140.87, 138.44, 137.92, 136.83, 136.10, 135.63, 133.97, 129.79, 128.08, 126.12, 125.57, 114.90, 103.41, 89.06, 86.66, 73.12, 63.79, 41.44, 41.01, 39.51, 23.74, 14.82. HRMS (ESI) m/z: $[M + H]^+$ calculated for $[C_{27}H_{29}N_4O_3S]^+$: 489.1955, found: 489.1961.

**(2*R*,3*S*,5*R*)-5-(4-Amino-5-(2-(2-methyl-5-(pyridin-4-yl)thiophen-3-yl)cyclopent-1-en-1-yl)-7*H*-pyrrolo[2,3-*d*]pyrimidin-7-yl)-2-(hydroxymethyl)-tetrahydrofuran-3-ol (1b)**

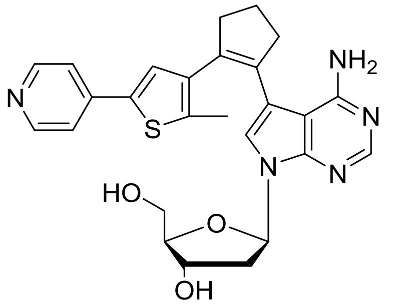


In a microwave vial (2*R*,3*S*,5*R*)-5-(4-amino-5-iodo-6-methyl-7*H*-pyrrolo[2,3-*d*]pyrimidin-7-yl)-2-(hydroxymethyl)tetrahydrofuran-3-ol (50 mg, 0.134 mmol), 4-(5-methyl-4-(2-(4,4,5,5-tetramethyl-1,3,2-dioxaborolan-2-yl)cyclopent-1-en-1-yl)thiophen-2-yl)pyridine (98 mg, 0,268 mmol), Pd(dppf)$Cl_2$ (6 mg, 6.7 µmol) and $Cs_2CO_3$ (217 mg, 0.67 mmol) were dissolved in acetonitrile (2 mL) and water (1 mL) under argon. The mixture was degassed 3 times and stirred at 120 °C for 1 h. The reaction mixture was absorbed on silica and purified by flash column chromatography (silica gel, DCM/MeOH 10:1 + 1% $NEt_3$) affording **1c** as yellowish solid in a yield of 56%.

$^1$H-NMR (300 MHz, $CD_3OD$): δ = 8.40 (d, J = 5.5 Hz, 2H), 7.99 (s, 1H), 7.41 (s, 1H), 7.41 (d, J = 4.9 Hz, 2H), 7.33 (s, 1H), 6.51 (dd, J = 8.1, 6.0 Hz, 1H), 4.51 (dt, J = 5.4, 2.5 Hz, 1H), 4.01 (dd, J = 6.1, 3.4 Hz, 1H), 3.82 – 3.66 (m, 2H), 2.98 – 2.88 (m, 4H), 2.68 – 2.56 (m, 1H), 2.31 (ddd, J = 13.2, 5.9, 2.6 Hz, 1H), 2.20 – 2.12 (m, 2H), 2.07 (s, 3H). $^{13}$C-NMR (125 MHz, $CD_3OD$): δ = 158.81, 152.11, 150.42, 150.38, 143.78, 139.66, 137.84, 136.92, 136.44, 134.87, 129.11, 122.63, 120.66, 114.61, 103.22, 89.06, 86.53, 73.10, 63.79, 41.44, 40.96, 39.47, 23.75, 14.96. HRMS (ESI) m/z: $[M - H]^-$ calculated for $[C_{26}H_{26}N_5O_3S]^-$: 488.1762, found: 488.1747.

**(2*R*,3*S*,5*R*)-5-(4-Amino-5-(2-(2-methyl-5-(naphthalen-2-yl)thiophen-3-yl)cyclopent-1-en-1-yl)-7*H*-pyrrolo[2,3-*d*]pyrimidin-7-yl)-2-(hydroxymethyl)tetrahydrofuran-3-ol (1d)**

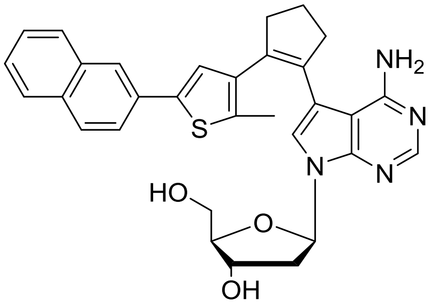


In a microwave vial (2*R*,3*S*,5*R*)-5-(4-amino-5-iodo-6-methyl-7*H*-pyrrolo[2,3-*d*]pyrimidin-7-yl)-2-(hydroxymethyl)tetrahydrofuran-3-ol (50 mg, 0.134 mmol), 4,4,5,5-tetramethyl-2-(2-(2-methyl-5-(naphthalen-2-yl)thiophen-3-yl)cyclopent-1-en-1-yl)-1,3,2-dioxaborolane (112 mg, 0,268 mmol), Pd(dppf)$Cl_2$ (6 mg, 6.7 µmol) and $Cs_2CO_3$ (217 mg, 0.67 mmol) were dissolved in acetonitrile (2 mL) and water (1 mL) under argon. The mixture was degassed 3 times and stirred at 120 °C for 1 h. The reaction mixture was absorbed on silica and purified by flash column chromatography (silica gel, DCM/MeOH 10:1) affording **1d** as yellowish solid in a yield of 97%.

$^1$H-NMR (300 MHz, $CD_3OD$): δ = 7.99 (s, 1H), 7.71 (d, J = 9.1 Hz, 4H), 7.49 – 7.35 (m, 3H), 7.33 (s, 1H), 7.12 (s, 1H), 6.51 (t, J = 6.8 Hz, 1H), 4.50 (s, 1H), 4.01 (s, 1H), 3.74 (ddd, J = 26.7, 12.1, 2.9 Hz, 2H), 2.88 (d, J = 7.1 Hz, 4H), 2.67 – 2.55 (m, 1H), 2.30 (dd, J = 12.9, 3.6 Hz, 1H), 2.16 – 2.07 (m, 2H), 2.04 (s, 3H). $^{13}$C-NMR (125 MHz, $CD_3OD$): δ = 158.15, 150.99, 150.18, 140.81, 137.00, 136.85, 136.51, 134.98, 133.88, 133.76, 132.95, 129.39, 128.83, 128.61, 127.50, 126.78, 126.09, 124.68, 124.15, 122.83, 115.25, 103.27, 89.08, 86.60, 73.08, 63.73, 41.51, 40.91, 39.44, 23.72, 14.90. HRMS (ESI) m/z: $[M + Na]^+$ calculated for $[C_{31}H_{30}N_4O_3SNa]^+$: 561.1931; found: 561.1929.

**(2*R*,3*S*,5*R*)-5-(4-Amino-5-(2-(2,5-dimethylthiophen-3-yl)cyclopent-1-en-1-yl)-6-methyl-7*H*-pyrrolo[2,3-*d*]pyrimidin-7-yl)-2-(hydroxymethyl)tetrahydrofuran-3-ol (2a)**

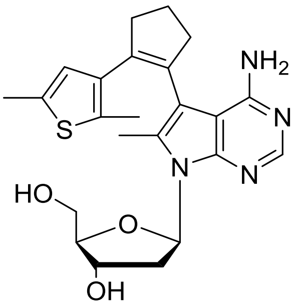


In a microwave vial (2*R*,3*S*,5*R*)-5-(4-amino-5-iodo-6-methyl-7*H*-pyrrolo[2,3-*d*]pyrimidin-7-yl)-2-(hydroxymethyl) tetrahydrofuran-3-ol (50 mg, 0.134 mmol), 2-(2,5-dimethylthiophen-3-yl)-4,4,5,5-tetramethyl-1,3,2-dioxaborolane (78 mg, 0,256 mmol), Pd(dppf)$Cl_2$ (6 mg, 6.7 µmol) and $Cs_2CO_3$ (217 mg, 0.67 mmol) were dissolved in acetonitrile (2 mL) and water (1 mL) under argon. The mixture was degassed 3 times and stirred at 120 °C for 1 h. The reaction mixture was absorbed on silica and purified by flash column chromatography (silica gel, DCM/MeOH 10:1) affording **2a** as yellowish solid in a yield of 52%.

$^1$H-NMR (300 MHz, $CD_3OD$): δ = 7.98 (s, 1H), 6.42 (dd, J = 7.9, 1.0 Hz, 1H), 6.34 – 6.26 (m, 1H), 4.62 – 4.55 (m, 1H), 4.09 – 4.05 (m, 1H), 3.89 (ddd, J = 12.3, 6.1, 2.3 Hz, 1H), 3.75 (dt, J = 12.3, 2.3 Hz, 1H), 3.12 – 2.68 (m, 6H), 2.26 (d, J = 3.3 Hz, 3H), 2.17 (s, 3H), 2.13 (d, J = 3.0 Hz, 3H), 2.12 – 2.07 (m, J = 7.4 Hz, 2H). $^{13}$C-NMR (125 MHz, $CD_3OD$): δ = 158.15, 150.71, 150.31, 140.68, 140.50, 136.30, 133.46, 132.66, 131.40, 127.10, 111.16, 103.98, 89.64, 87.08, 73.91, 64.27, 40.96, 40.37, 39.17, 24.20, 14.93, 14.49, 11.13. HRMS (ESI) m/z: $[M + H]^+$ calculated for $[C_{23}H_{29}N_4O_3S]^+$: 441.1955, found: 441.1949.

**(2*R*,3*S*,5*R*)-5-(4-Amino-6-methyl-5-(2-(2-methyl-5-phenylthiophen-3-yl)cyclopent-1-en-1-yl)-7*H*-pyrrolo[2,3-*d*]pyrimidin-7-yl)-2-(hydroxymethyl)tetrahydrofuran-3-ol (2c) [2]**

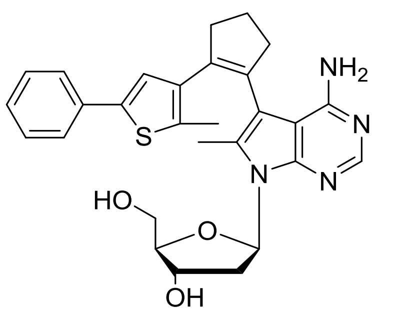


In a microwave vial (2*R*,3*S*,5*R*)-5-(4-amino-5-iodo-6-methyl-7*H*-pyrrolo[2,3-*d*]pyrimidin-7-yl)-2-(hydroxymethyl) tetrahydrofuran-3-ol (50 mg, 0.134 mmol), 4,4,5,5-tetramethyl-2-(2-(2-methyl-5-phenylthiophen-3-yl)cyclopent-1-en-1-yl)-1,3,2-dioxaborolane (94 mg, 0,256 mmol), Pd(dppf)$Cl_2$ (6 mg, 6.7 µmol) and $Cs_2CO_3$ (217 mg, 0.67 mmol) were dissolved in acetonitrile (2 mL) and water (1 mL) under argon. The mixture was degassed 3 times and stirred at 120 °C for 1 h. The reaction mixture was absorbed on silica and purified by flash column chromatography (silica gel, DCM/MeOH 10:1) affording **2b** as yellowish solid in a yield of 36%.

$^1$H-NMR (300 MHz, $CD_3OD$): δ = 7.98 (s, 1H), 7.41 – 7.25 (m, 5H), 7.01 (d, J = 14.4 Hz, 1H), 6.31 (dt, J = 9.4, 6.1 Hz, 1H), 4.61 – 4.54 (m, 1H), 4.07 (s, 1H), 3.93 – 3.84 (m, 1H), 3.79 – 3.70 (m, 1H), 3.10 – 2.92 (m, 4H), 2.87 – 2.75 (m, 2H), 2.18 (d, J = 1.4 Hz, 3H), 2.17 – 2.10 (m, 2H), 2.08 (d, J = 3.0 Hz, 3H). $^{13}$C-NMR (125 MHz, $CD_3OD$): δ = 158.14, 150.80, 150.43, 140.81, 140.37, 136.89, 135.69, 135.58, 133.54, 131.54, 129.81, 128.09, 126.17, 125.18, 111.07, 103.91, 89.62, 87.05, 73.88, 64.29, 40.89, 40.33, 39.05, 24.25, 14.73, 11.25. HRMS (ESI) m/z: $[M + H]^+$ calculated for $[C_{28}H_{31}N_4O_3S]^+$: 503.2111, found: 503.2114.

**(2*R*,3*S*,5*R*)-5-(4-Amino-6-methyl-5-(2-(2-methyl-5-(pyridin-4-yl)thiophen-3-yl)cyclopent-1-en-1-yl)-7*H*-pyrrolo[2,3-*d*]pyrimidin-7-yl)-2-(hydroxymethyl)tetrahydrofuran-3-ol (2b) [2]**

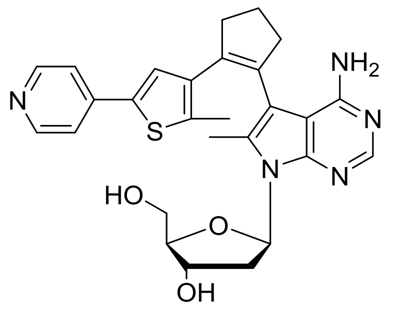


In a microwave vial (2*R*,3*S*,5*R*)-5-(4-amino-5-iodo-6-methyl-7*H*-pyrrolo[2,3-*d*]pyrimidin-7-yl)-2-(hydroxymethyl) tetrahydrofuran-3-ol (50 mg, 0.134 mmol), 4-(5-methyl-4-(2-(4,4,5,5-tetramethyl-1,3,2-dioxaborolan-2-yl)cyclopent-1-en-1-yl)thiophen-2-yl)pyridine (94 mg, 0,256 mmol), Pd(dppf)$Cl_2$ (6 mg, 6.7 µmol) and $Cs_2CO_3$ (217 mg, 0.67 mmol) were dissolved in acetonitrile (2 mL) and water (1 mL) under argon. The mixture was degassed 3 times and stirred at 120 °C for 1 h. The reaction mixture was absorbed on silica and purified by flash column chromatography (silica gel, DCM/MeOH 10:1) affording **2c** as yellowish solid in a yield of 38%.

$^1$H-NMR (300 MHz, $CD_3OD$): δ = 8.41 (d, J = 5.1 Hz, 2H), 7.98 (s, 1H), 7.45 – 7.40 (m, 2H), 7.37 (d, J = 2.8 Hz, 1H), 6.31 (ddd, J = 8.1, 5.8, 2.0 Hz, 1H), 4.58 (d, J = 5.8 Hz, 1H), 4.10 – 4.03 (m, 1H), 3.92 – 3.83 (m, 1H), 3.78 – 3.69 (m, 1H), 3.08 – 2.78 (m, 6H), 2.27 – 2.20 (m, 2H), 2.19 (d, J = 3.2 Hz, 3H), 2.10 (d, J = 4.6 Hz, 3H). $^{13}$C-NMR (125 MHz, $CD_3OD$): δ = 158.05, 150.84, 150.55, 150.44, 143.75, 139.77, 139.40, 137.85, 136.93, 134.48, 131.62, 128.57, 120.70, 110.80,103.76, 89.65, 87.07, 73.87, 64.30, 40.88, 40.28, 39.00, 24.19, 14.91, 11.25. HRMS (ESI) m/z: $[M + H]^+$ calculated for $[C_{27}H_{30}N_5O_3S]^+$: 504.2064, found: 504.2063.

**(2*R*,3*S*,5*R*)-5-(4-Amino-6-methyl-5-(2-(2-methyl-5-(naphthalen-2-yl)thiophen-3-yl)cyclopent-1-en-1-yl)-7*H*-pyrrolo-[2,3-*d*]pyrimidin-7-yl)-2-(hydroxymethyl) tetrahydrofuran-3-ol (2d)**

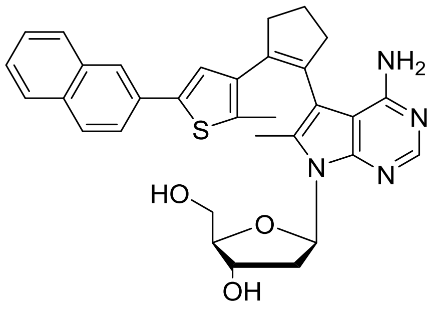


In a microwave vial (2*R*,3*S*,5*R*)-5-(4-amino-5-iodo-6-methyl-7*H*-pyrrolo[2,3-*d*]pyrimidin-7-yl)-2-(hydroxymethyl) tetrahydrofuran-3-ol (50 mg, 0.134 mmol), 4,4,5,5-tetramethyl-2-(2-(2-methyl-5-(naphthalen-2-yl)thiophen-3-yl)cyclopent-1-en-1-yl)-1,3,2-dioxaborolane (94 mg, 0,256 mmol), Pd(dppf)$Cl_2$ (6 mg, 6.7 µmol) and $Cs_2CO_3$ (217 mg, 0.67 mmol) were dissolved in acetonitrile (2 mL) and water (1 mL) under argon. The mixture was degassed 3 times and stirred at 120 °C for 1 h. The reaction mixture was absorbed on silica and purified by flash column chromatography (silica gel, DCM/MeOH 10:1) affording **2d** as yellowish solid in a yield of 55%.

$^1$H-NMR (300 MHz, $CD_3OD$): δ = 8.00 (s, 1H), 7.78 – 7.70 (m, 4H), 7.52 – 7.34 (m, 3H), 7.10 (d, J = 13.6 Hz, 1H), 6.36 – 6.26 (m, 1H), 4.57 (d, J = 5.7 Hz, 1H), 4.07 (d, J = 1.6 Hz, 1H), 3.88 (dt, J = 12.3, 2.2 Hz, 1H), 3.73 (dd, J = 12.3, 2.4 Hz, 1H), 3.14 – 2.61 (m, 6H), 2.16 (s, 3H), 2.15 – 2.09 (m, 2H), 2.08 (d, J = 1.6 Hz, 3H). $^{13}$C-NMR (125 MHz, $CD_3OD$): δ =158.16, 150.83, 150.47, 140.71, 140.29, 137.00, 136.12, 135.04, 133.93, 133.55, 132.96, 131.54, 129.44, 128.81, 128.65, 127.47, 126.81, 125.68, 124.73, 124.16, 111.08,103.94, 89.65, 87.09, 73.91, 64.34, 40.86, 40.36, 38.99, 24.26, 14.84, 11.27. HRMS (ESI) m/z: [M + H]$^+$ calculated for $[C_{32}H_{33}N_4O_3S]^+$: 553.2268, found: 553.2267.

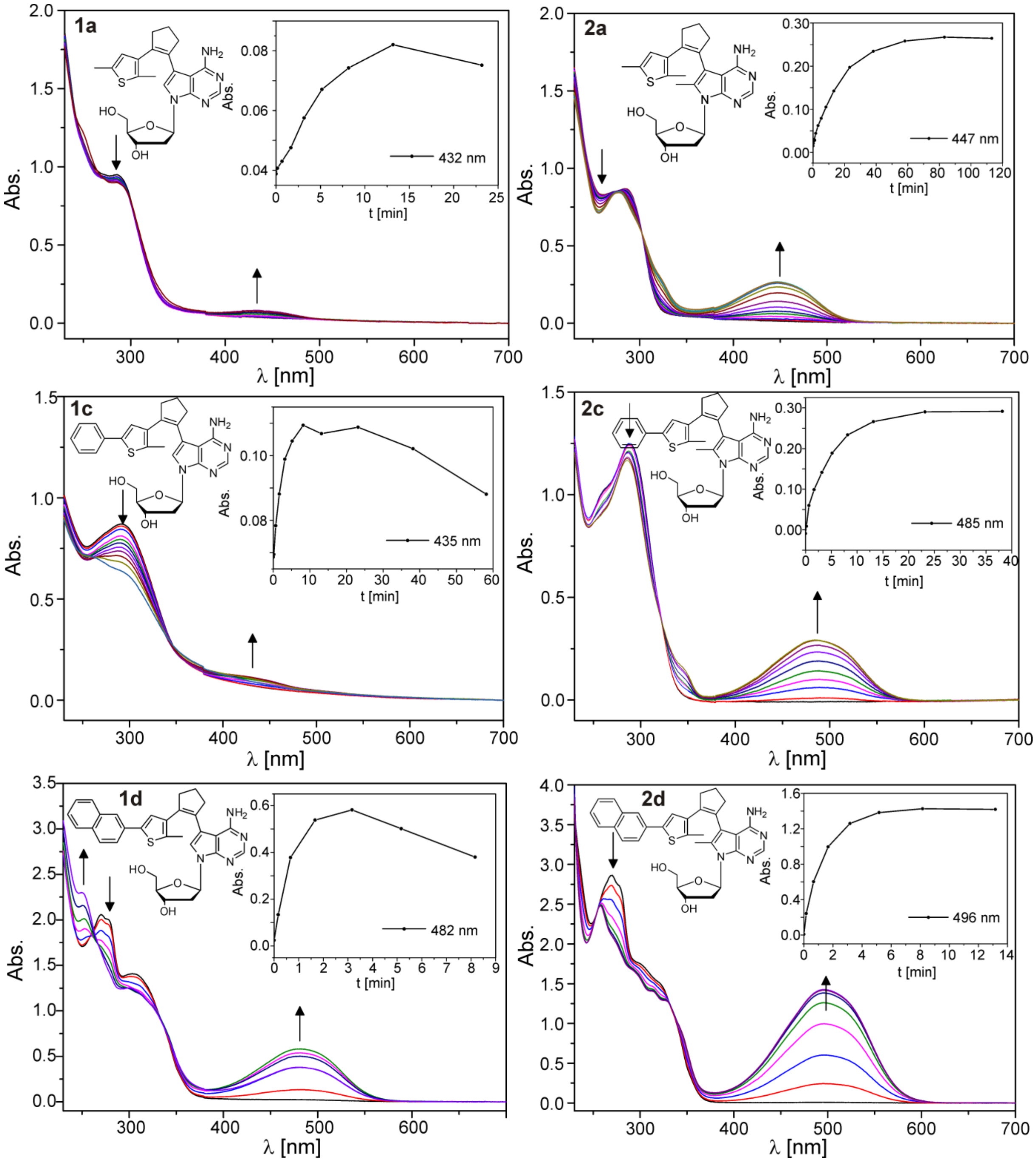

**Figure S1:** Spectral changes of the 7-deazaadenosine photoswitches with one (**1a,c,d**) and two (**2a,c,d**) methyl groups upon irradiation with UV light. A 60 µM solution of the compound in acetonitrile was irradiated for the indicated time intervals (see inset) with UV light (366 nm) and after each interval a UV–vis absorption spectrum was recorded.

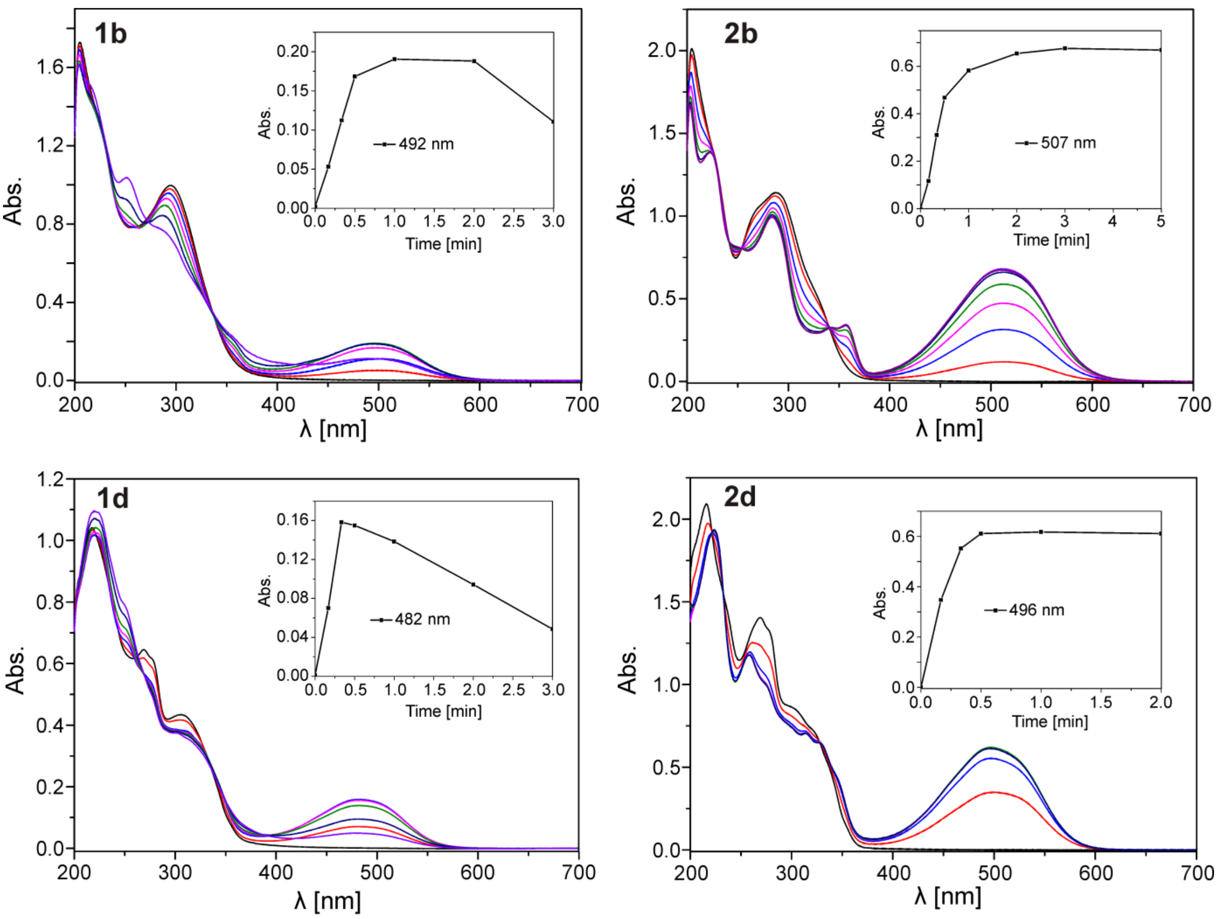


| R | $\lambda_{max,vis}$ (**1b,d**) | $A_{\lambda max,vis}/A_{280nm}$ (**1b,d**) | $\lambda_{max,vis}$ (**2b,d**) | $A_{\lambda max,vis}/A_{280nm}$ (**2b,d**) | Ratio*[1] | $\Delta\lambda_{21}$*[2] |
|---|---|---|---|---|---|---|
| **b** | 492 nm | 0,15 | 507 nm | 0,68 | 15 nm | 4,6 |
| **d** | 482 nm | 0,36 | 496 nm | 0,63 | 14 nm | 1,75 |

**Figure S2:** Spectral changes of 7-deazaadenosine photoswitches with one (**1b, d**) and two (**2b, d**) methyl groups upon irradiation with UV light. A 60 µM solution of the compound in ethanol was irradiated for the indicated time intervals (see inset) with UV light (310 nm, LED) and after each interval a UV–vis absorption spectrum was recorded.

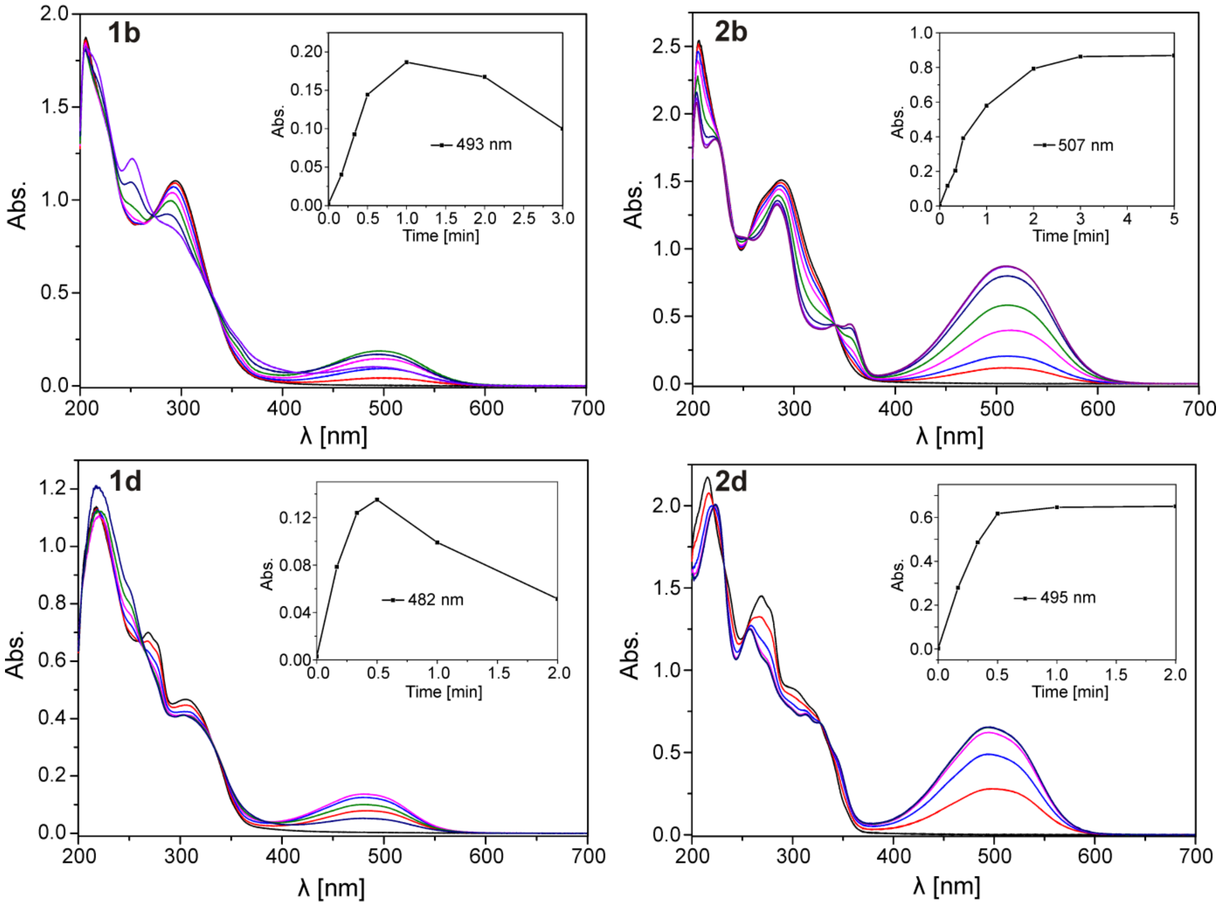


| R | $\lambda_{max,vis}$ (**1b,d**) | $A_{\lambda max,vis}/A_{280nm}$ (**1b,d**) | $\lambda_{max,vis}$ (**2b,d**) | $A_{\lambda max,vis}/A_{280nm}$ (**2b,d**) | Ratio*[1] | $\Delta\lambda_{21}$*[2] |
|---|---|---|---|---|---|---|
| **b** | 493 nm | 0.14 | 507 nm | 0.67 | 14 nm | 4.8 |
| **d** | 482 nm | 0.34 | 495 nm | 0.65 | 13 nm | 1.9 |

**Figure S3:** Spectral changes of 7-deazaadenosine photoswitches with one (**1b, d**) and two (**2b, d**) methyl groups upon irradiation with UV light. A 60 µM solution of the compound in methanol was irradiated for the indicated time intervals (see inset) with UV light (310 nm, LED) and after each interval a UV–vis absorption spectrum was recorded.

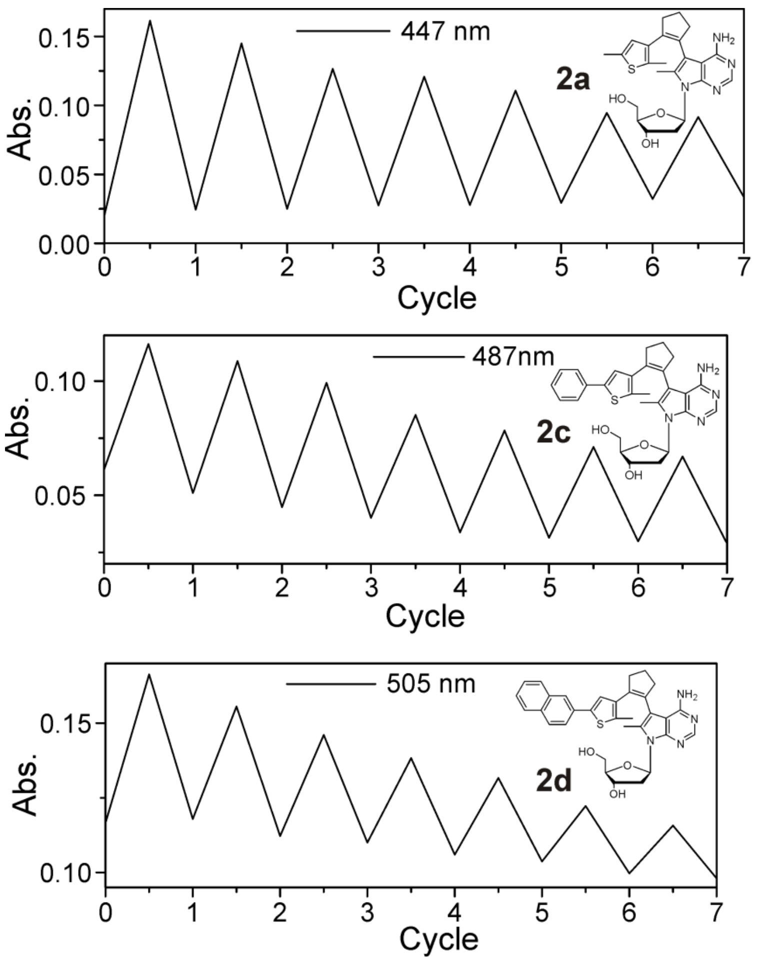


**Figure S4:** Reversibility measurements of 7-deazaadenosine photoswitches with two methyl groups (**2a,c,d**). Switches with one methyl group (**1a,c,d**) were not measured, because of low photochromicity and observed instability against extensice UV-ligth irradiation. A 60 µM solution of the compound in acetonitrile was irradiated with UV light until the photostationary state was reached. After recording the UV–vis spectrum, the solution was irradiated with vis light for the same time. This procedure was repeated 7 times.

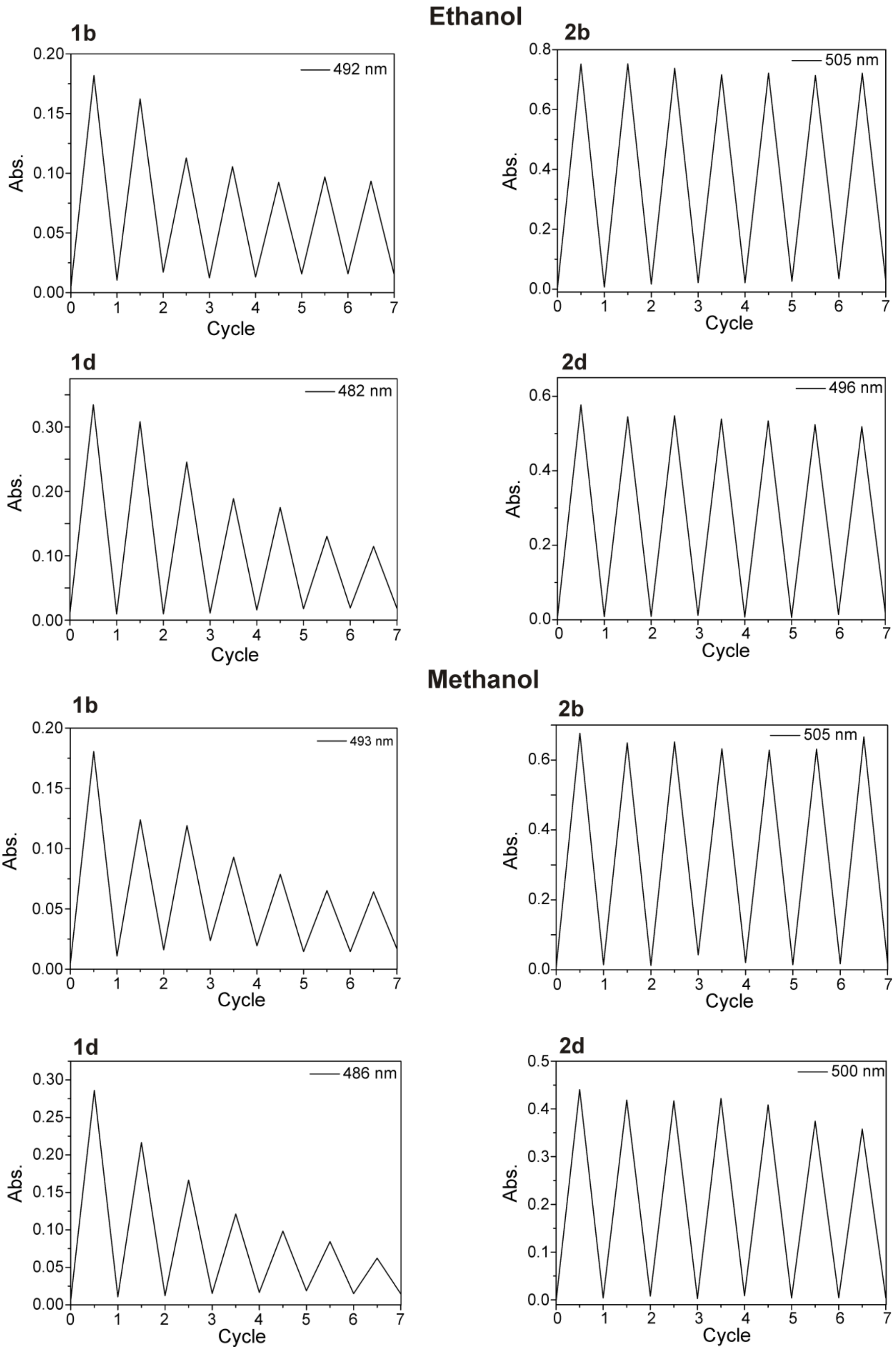


**Figure S5:** Reversibility measurements of 7-deazaadenosine photoswitches with one (**1b, d)** and two methyl groups (**1b, d**) in different solvents (ethanol, methanol). A 60 µM solution of the compound in ethanol/methanol was irradiated with UV light until the (pseudo-) photostationary state was reached. After recording the UV–vis spectrum, the solution was irradiated with vis light for the same time. This procedure was repeated 7 times.

**A**

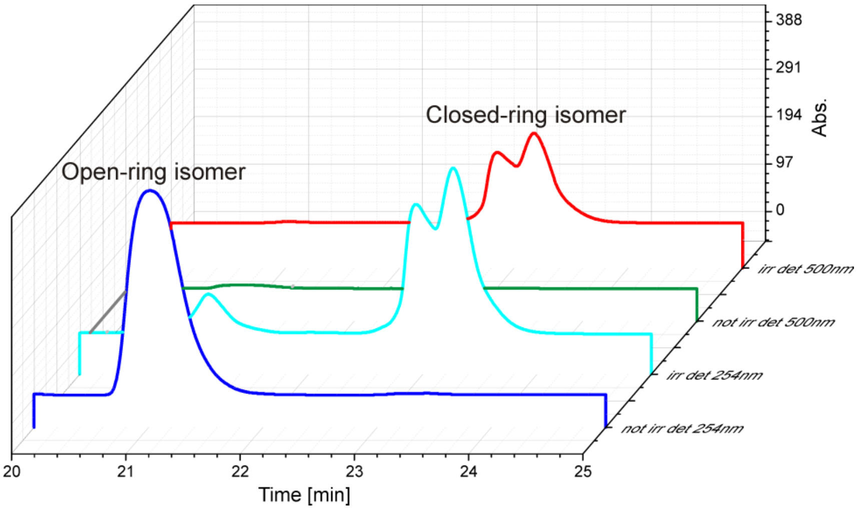
Closed-ring isomer
Open-ring isomer
388
291
194
97
0
Abs.
irr det 500nm
not irr det 500nm
irr det 254nm
not irr det 254nm
20
21
22
23
24
25
Time [min]

**B**

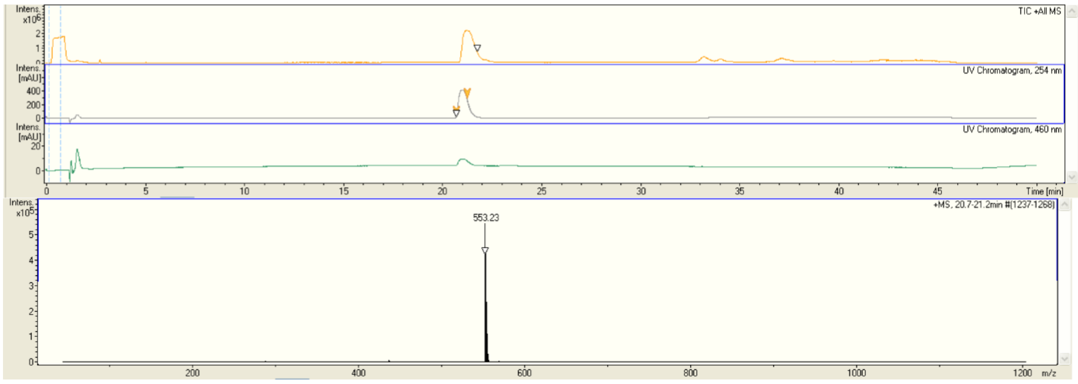
TIC +All MS
UV Chromatogram, 254 nm
UV Chromatogram, 460 nm
Time [min]
553.23
+MS, 20.7-21.2min #(1237-1268)
m/z

**C**

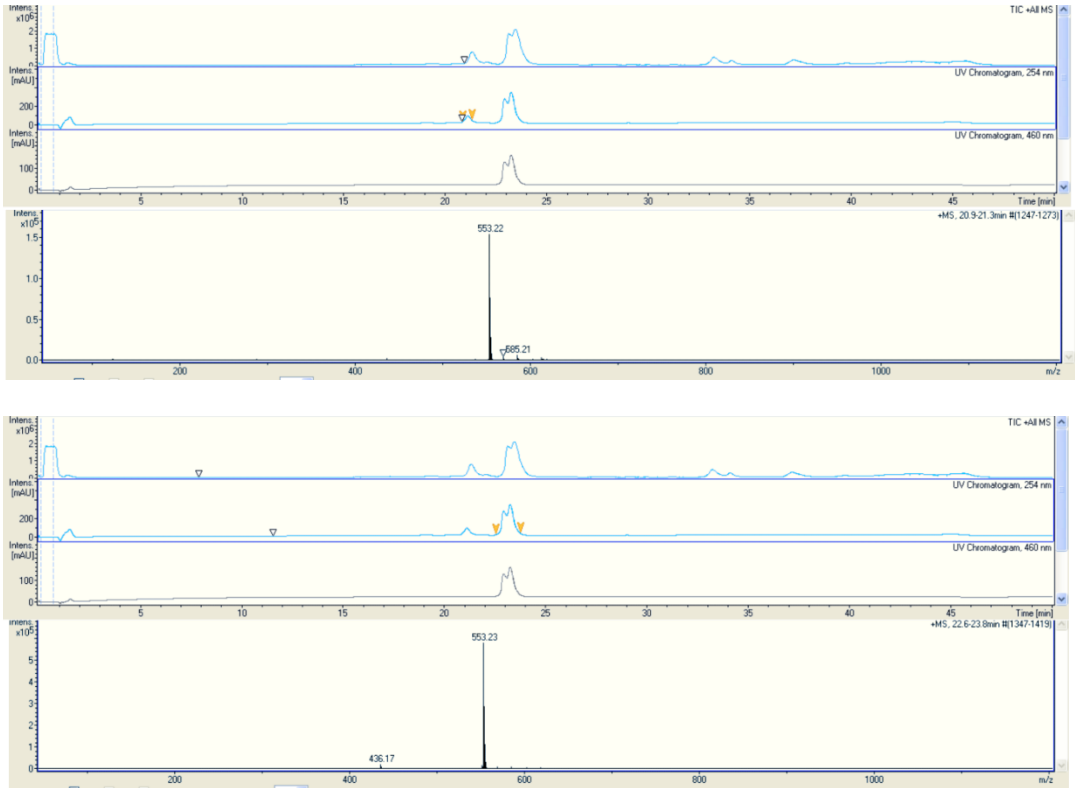
TIC +All MS
UV Chromatogram, 254 nm
UV Chromatogram, 460 nm
Time [min]
553.22
585.21
+MS, 20.9-21.3min #(1247-1273)
553.23
436.17
+MS, 22.6-23.8min #(1347-1419)
m/z

**D**

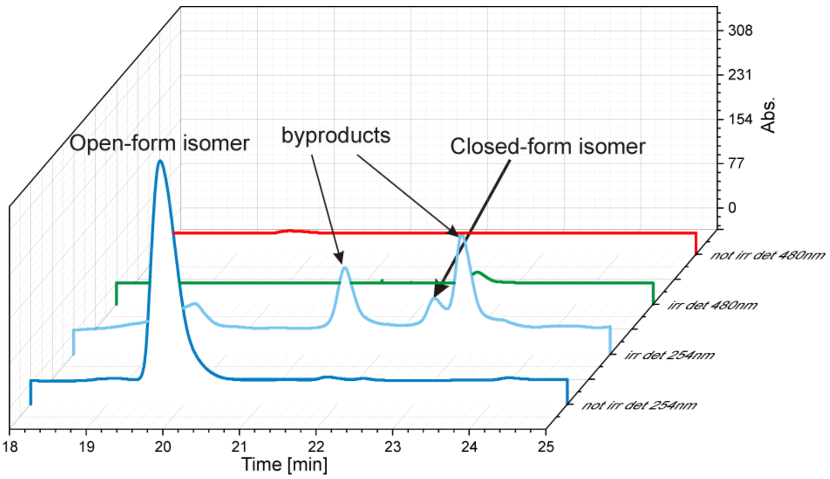
Open-form isomer
byproducts
Closed-form isomer
Abs.
308
231
154
77
0
not irr det 480nm
irr det 480nm
irr det 254nm
not irr det 254nm
18
19
20
21
22
23
24
25
Time [min]

**E**

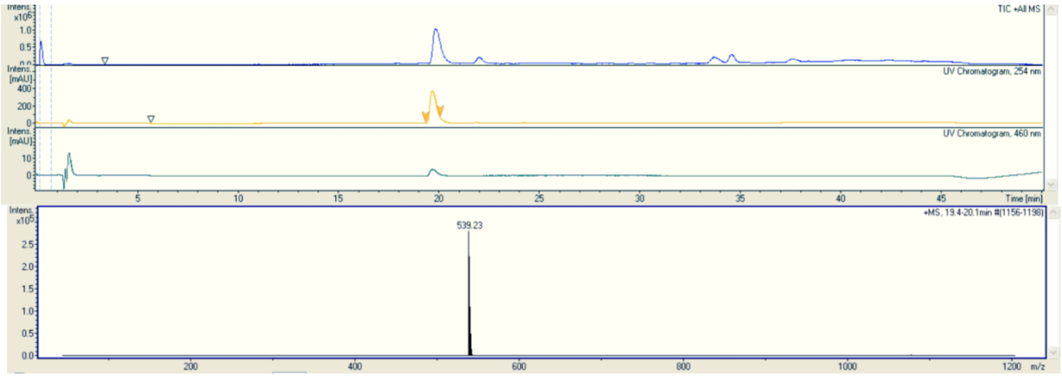
TIC +All MS
UV Chromatogram, 254 nm
UV Chromatogram, 460 nm
539.23
Time [min]
m/z

**F**

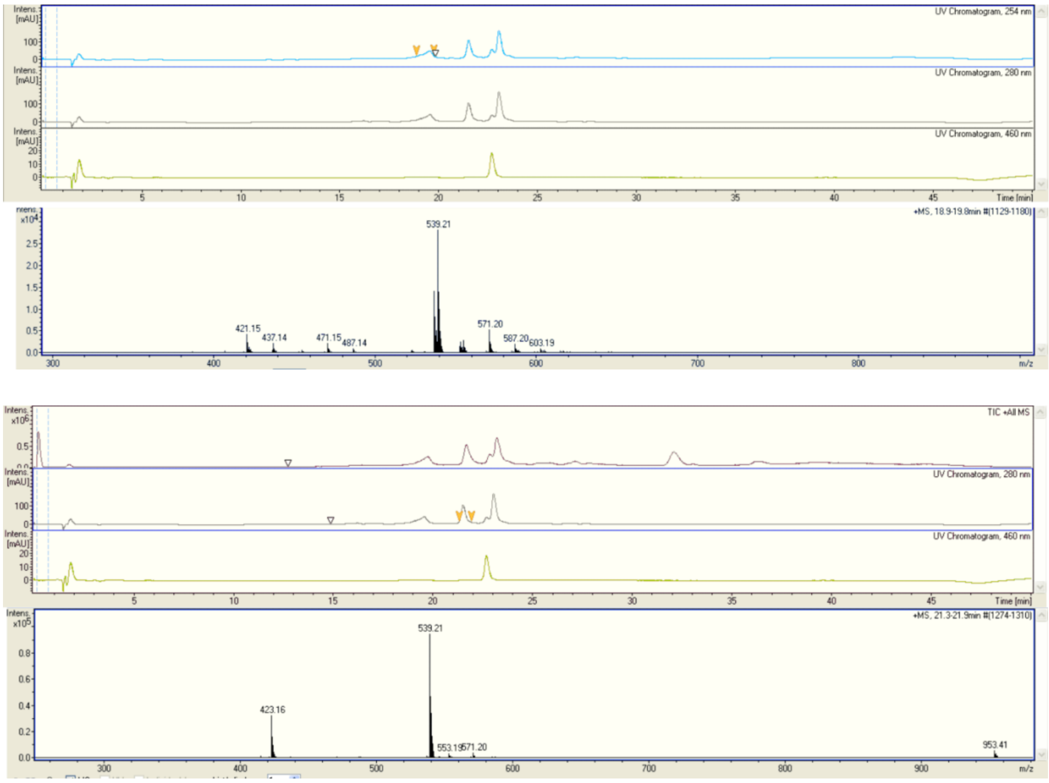
UV Chromatogram, 254 nm
UV Chromatogram, 280 nm
UV Chromatogram, 460 nm
539.21
421.15
437.14
471.15
487.14
571.20
587.20
603.19
TIC +All MS
423.16
553.19
953.41
Time [min]
m/z

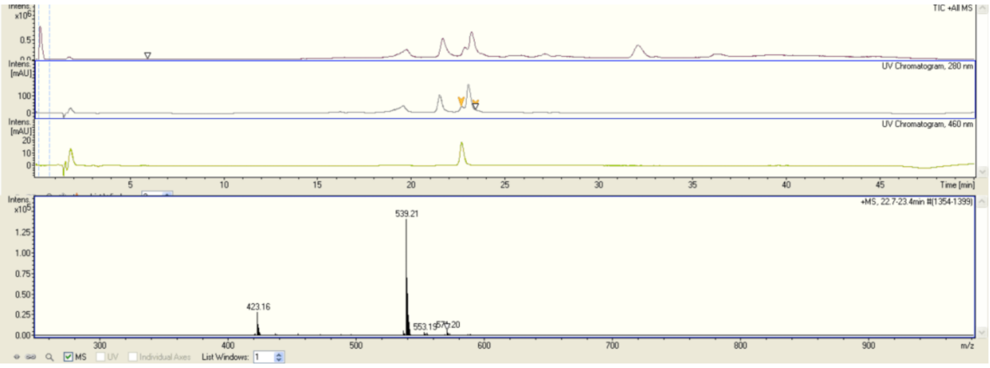


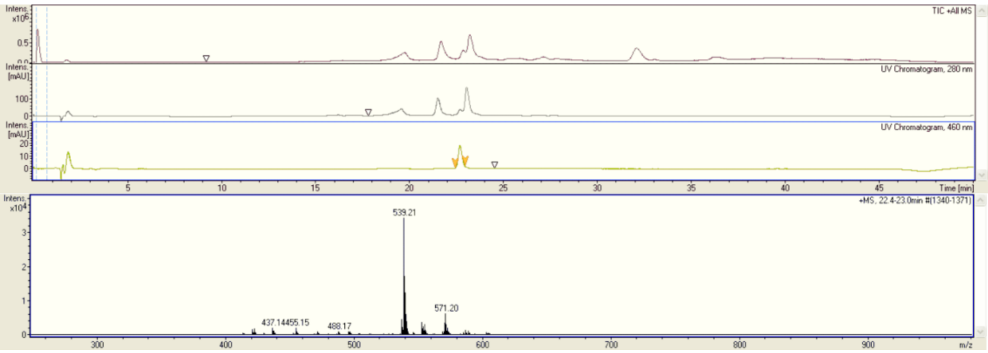


**Figure S6:** LC–MS analysis of isomerization and degradation products of **1d** and **2d**. **A:** HPLC chromatogram of compound **2d** before and after extensive irradiation with UV light (366 nm) detected at 254 nm and around the maximum of the closed ring isomer (500 nm). **B, C:** TIC (total ion current) and UV chromatograms recorded at 254 and 460 nm for compound **2d** before (**B**) and after extensive irradiation (**C**) with UV light (366 nm). The arrows on the right and left side at the corresponding peak indicate that the recorded mass spectrum below corresponds to this peak. **D:** HPLC chromatogram of compound **1d** before and after extensive irradiation with UV light (366nm) detected at 254 nm and around the maximum of the closed ring isomer (480 nm). **E, F:** TIC and UV chromatograms recorded at 254 and 460 nm for compound **1d** before (**E**) and after extensive irradiation (**F**) with UV light (366 nm).

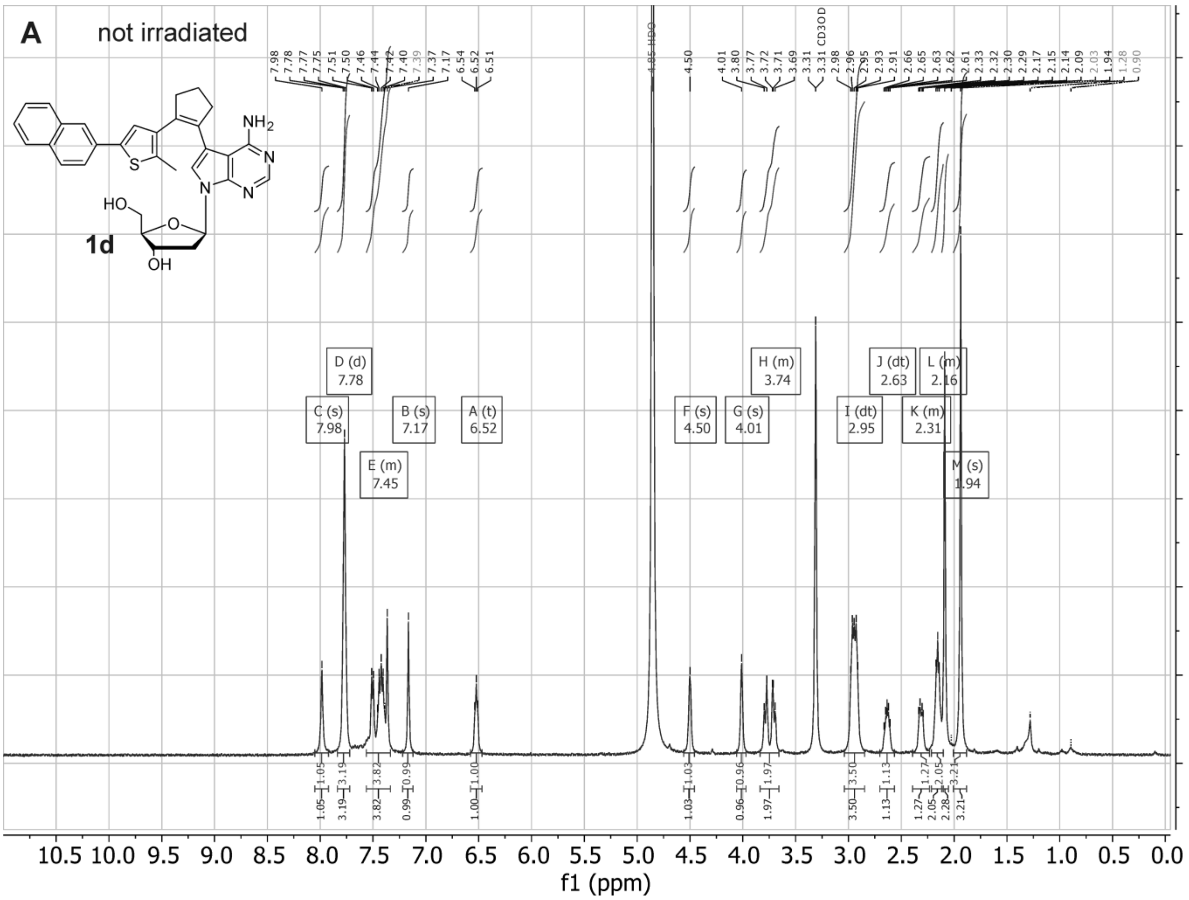
A
not irradiated
1d
NH2
HO
OH
f1 (ppm)

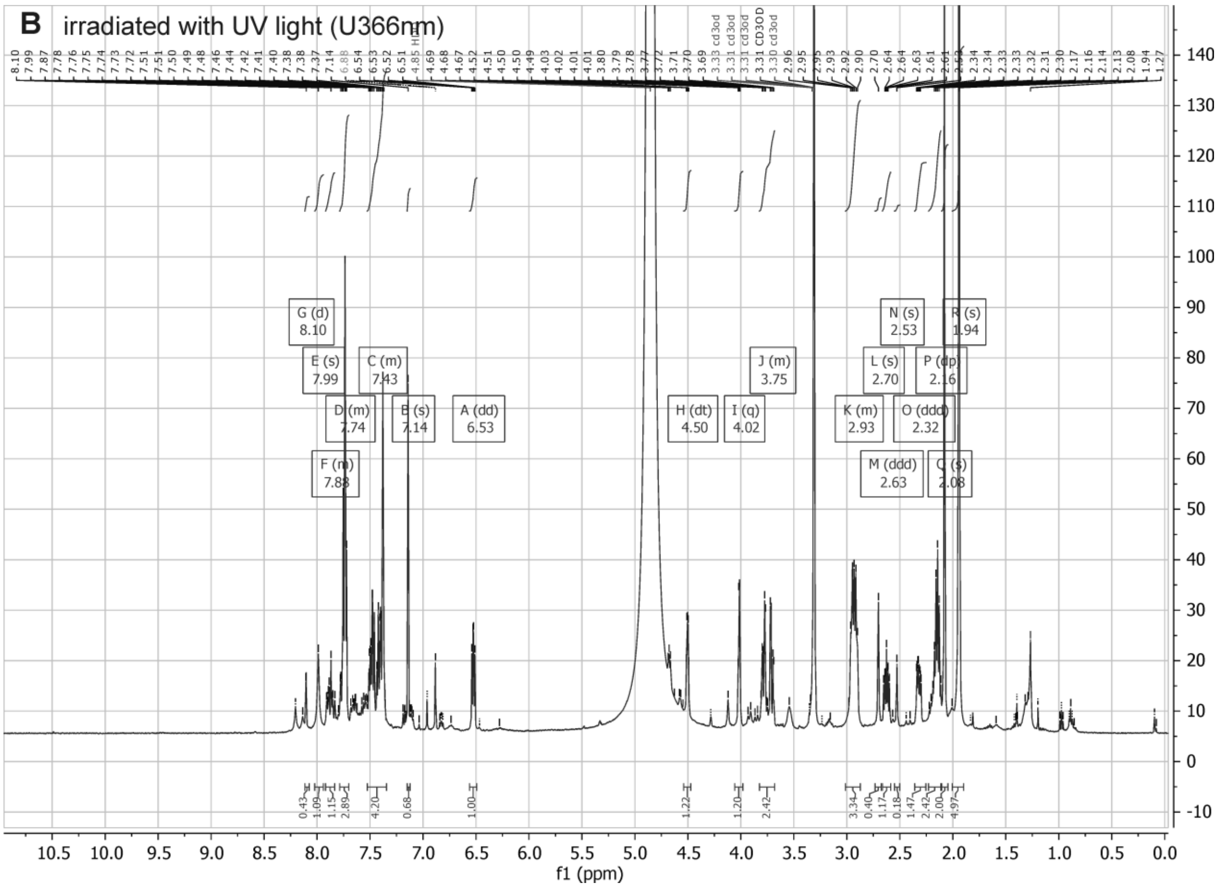
B
irradiated with UV light (U366nm)
f1 (ppm)

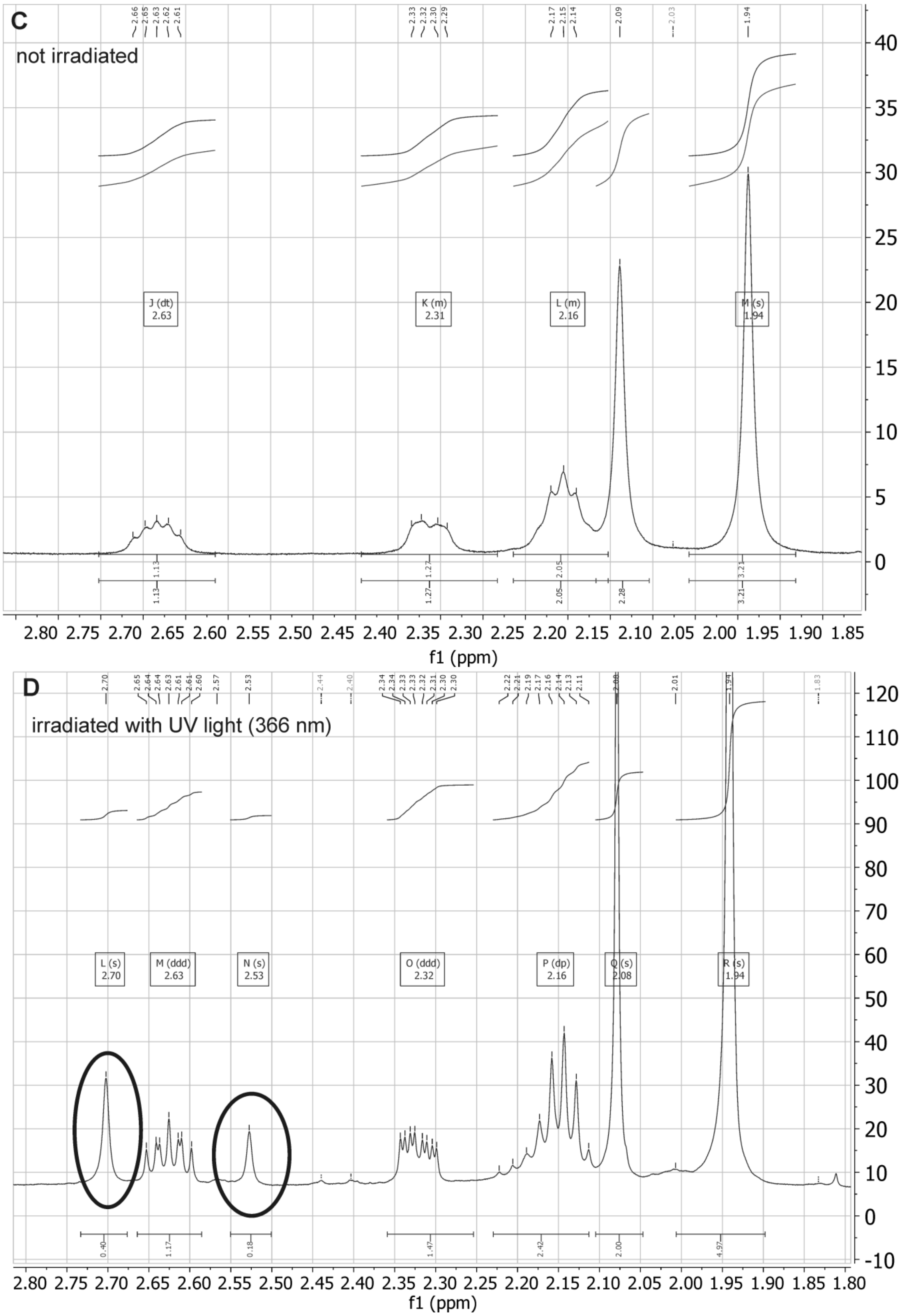


**Figure S7: A:** NMR spectrum of compound **1d** before irradiation with UV light (366 nm). **B:** NMR spectrum of compound **1d** after extensive irradiation with UV light (366 nm). **C:** NMR spectrum of compound **1d** before irradiation with UV light zoomed into the range of 1.85 ppm – 2.85 ppm. **D:** NMR spectrum of compound **1d** after extensive irradiation with UV light zoomed into the range of 1.85 ppm – 2.85 ppm.

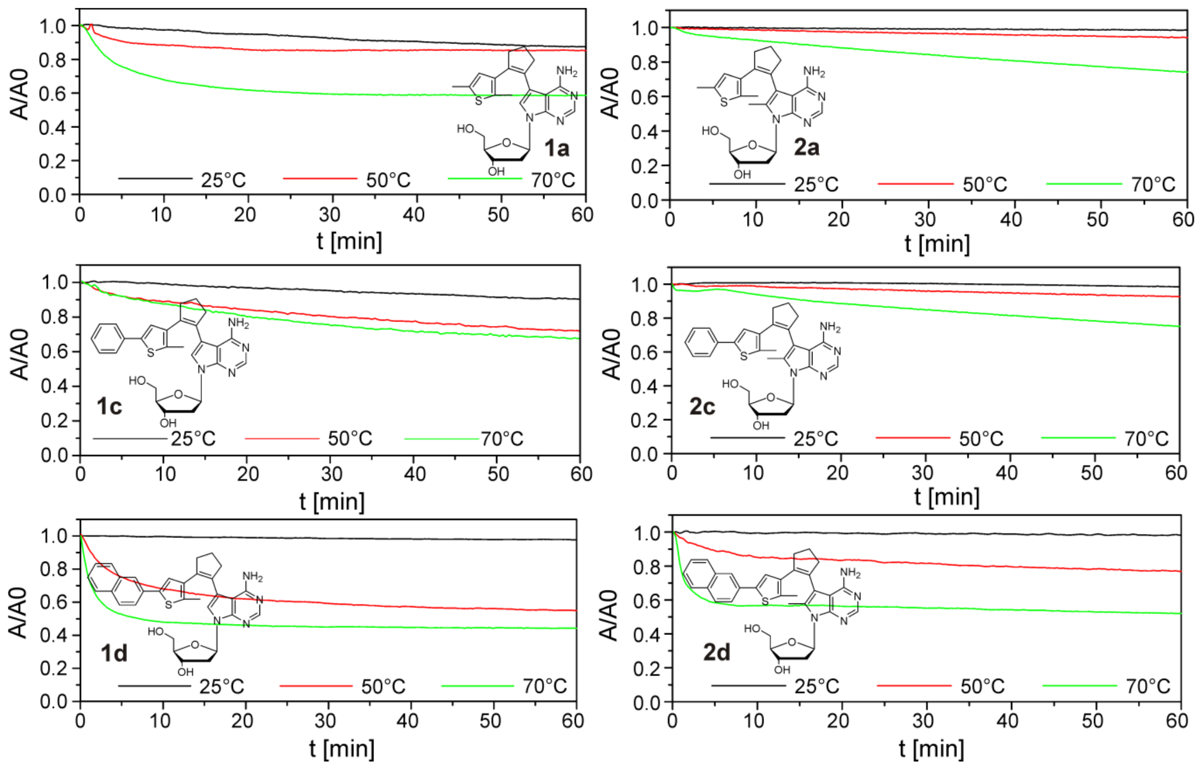


**Figure S8:** Thermostability measurements of the 7-deazaadenosine nucleosides with one (**1,a,c,d**) and two (**2a,c,d**) methyl groups. A 60 µM solution of the compound in acetonitrile was irradiated with UV light until the (pseudo)-photostationary state was reached. Then UV irradiation was switched off and the absorption at the visible light maximum of the closed ring form was measured over 1 h.

## Supporting references


1. Still, W. C.; Kahn, M.; Mitra, A. *J. Org. Chem.* **1978,** *43*, 2923-2925. doi: 10.1021/jo00408a041.
2. Singer, M.; Jäschke, A. *J. Am. Chem. Soc.* **2010,** *132*, 8372-8377. doi: 10.1021/ja1024782.
3. Erko, F. G.; Cseh, L.; Berthet, J.; Mehl, G. H.; Delbaere, S. *Dyes Pigm.* **2015,** *115*, 102-109. doi: 10.1016/j.dyepig.2014.12.015.
4. Goldstein, D. M.; Brameld, K. A. Tyrosine kinase inhibitors. US8673925 B1, 9 Apr. 2013, 2013.
